# Patients’ and health professionals’ research priorities for chronic pain associated with inflammatory bowel disease: a co-produced sequential mixed methods Delphi consensus study

**DOI:** 10.1136/bmjgast-2024-001483

**Published:** 2024-09-12

**Authors:** Morris Gordon, Vassiliki Sinopoulou, Roxana Mardare, Mansour Abdulshafea, Ciaran Grafton-Clarke, Jessica Vasiliou

**Affiliations:** 1 University of Central Lancashire, Preston, UK; 2 Blackpool Victoria Hospital, Blackpool, UK; 3 Great Ormond Street Hospital for Children NHS Foundation Trust, London, London, UK; 4 University of East Anglia, Norwich, UK; 5 Crohn's & Colitis UK, Hatfield, Hertfordshire, UK

**Keywords:** IBD, ABDOMINAL PAIN, HEALTH SERVICE RESEARCH

## Abstract

**Objective:**

Chronic pain in inflammatory bowel disease (IBD) is common and detrimental to quality of life. Recent Cochrane reviews identified a multitude of randomised controlled trial interventions, but the certainty of the findings is low or very low. We set out to reach a patient and professional co-produced Delphi consensus on treatment priorities, key outcomes and propose a model for understanding our findings.

**Methods:**

An online survey was co-produced with Crohn’s and Colitis UK and sent to patients and healthcare professionals in two phases, for prioritisation of treatments and outcome measures. Phase three consisted of four online group interviews, where patients and healthcare professionals discussed the rationale of their choices. Transcripts were combined with the free text data from the Delphi surveys and analysed through a three-phase qualitative technique.

**Results:**

The phase 1 survey was completed by 128 participants (73 patients, 3 carers and 53 health professionals). Diet was the top priority for both patients (n=26/73, 36.1%) and healthcare professionals (n=29/52, 56.9%). Phase 2 was completed by 68 participants. FODMAP (fermentable oligosaccharides, disaccharides, monosaccharides and polyols) diet, stress management therapy and relaxation therapy were the top three consensus priorities. Phase 3 group interviews were attended by 13 patients and 5 healthcare professionals. Key themes included: The patient as an individual, beliefs and experiences, disease activity influencing therapy choice, accessibility barriers and quality of life.

**Conclusion:**

Low FODMAP diet, followed by psychological therapies were the highest-rated research priorities for healthcare professionals and patients. Funding bodies and researchers should consider these findings, alongside the model for understanding our findings, when making research decisions.

**WHAT IS ALREADY KNOWN ON THIS TOPIC**

**WHAT THIS STUDY ADDS**

- We reached a patient and professional consensus on treatment priorities and key outcomes for the treatment of pain in IBD and present a model for understanding our findings.

**HOW THIS STUDY MIGHT AFFECT RESEARCH, PRACTICE OR POLICY**

- Low FODMAP diet and psychological therapies are the treatments that should be prioritised among the multitude of trialled treatments, in order to reach high certainty conclusions on their efficacy and safety, according to patients and professionals.

## Introduction

 The prevalence of chronic pain (CP) in patients with inflammatory bowel disease (IBD) has been reported as high as 48% in outpatients and 38% in hospital-based cohorts with a considerable impact on all aspects of quality of life and higher costs and utilisation of healthcare.^1^,^3^

Mechanisms of CP in IBD are intricate and include mechanical causes like strictures or fistulae, small bowel bacterial overgrowth, post-surgical pain, visceral hypersensitivity, gut dysmotility, dysregulated pain signalling and psychological and social factors.^4^ There is increasing momentum for a biopsychosocial model that views CP in IBD as an interaction between inflammation and sensory pathways.^5^

Another cause for chronic pain in IBD could be the coexistence with irritable bowel syndrome (IBS), now referred to as IBD-IBS. A systematic review conducted on 13 studies and 1703 patients concluded that symptoms compatible with IBS are more common in patients with IBD than in healthy individuals.^6^ IBS was present in 44% of patients with IBD with active disease, but also in 35% of patients with IBD in remission.

Despite chronic pain in IBD being common and deleterious to quality of life, the treatment choices are scarce. Two Cochrane reviews on abdominal pain in ulcerative colitis (UC) and Crohn’s disease (CD) showed that even though a large number of treatments have been researched in randomised controlled trials (RCTs), the evidence for efficacy and safety remains of low and very low certainty.^7 8^ The reviews included 5 UC RCTs, mounting to 360 randomised patients and 14 CD RCTs including 743 randomised participants undergoing in total 14 interventions that have been or are currently being trialled at the RCT level. The number of RCTs trialling the same therapy was between 1 and 2, and there was heterogeneity in terms of outcome measures and assessment, causing the certainty of all results on efficacy and safety to be very low, or low at best.

Our aim was to reach a patient and professional co-produced Delphi consensus on treatment intervention priorities and key outcomes, between all the interventions and outcomes that have been used in RCTs and to present a model for understanding our findings.

## Methods

On completion of the two Cochrane systematic reviews, our team discussed dissemination possibilities with Crohn’s and Colitis UK (CCUK). In collaboration with them, we organised pre-planning consultation workshops which were attended by patients and one of CCUK representatives in charge of this project (JV). Via those workshops, it was co-decided that there was a need for research and evidence prioritisation for patients and healthcare professionals and the present study was planned.

This study employed a modified Delphi approach to reach a consensus on research priorities.^9^ It was decided that as well as a final phase of panellist confirmation of research priorities, an additional element of online workshops would be included. This was to achieve an understanding as to why the priorities were identified.

### Participants

Participants were patients and healthcare professionals who were invited to participate through the CCUK charity research- interested database (approximately 2000 patients and people) and British Society of Gastroenterology databases, respectively, by email and online advertisement. This study excluded children and young people under the age of 18. The project had ethical and research and development approval from the University of Central Lancashire Ethics board on 02 April 2020, application number Health 0050.

### Data collection

This study was carried out prospectively, in three Delphi phases comprised of an online survey in two parts (phase 1 and 2) and four online workshops (phase 3).

The modified Delphi process was used in phases 1 and 2 (with initial potential for further phases). This is a systematic and structured process designed to help a group of experts reach consensus, combining anonymous, iterative surveys to provide focus and prioritisation on a given problem. In line with Delphi methods, if consensus had not been reached and significant areas of misalignment existed, further phases would have been running. As this was not the case, the study proceeded directly.

In phase 1, a fully anonymised online survey was sent out to patients and healthcare professionals asking them to prioritise the interventions and outcomes used in RCTs up until the time of the survey, as these were identified by the two Cochrane systematic reviews.^7 8^ After filling in a consent form, participants followed a fully anonymised link to the survey. The online questionnaire was delivered through surveys.ac.uk, with a Likert scale from 1 to 7 to indicate the importance of each item (highest=1, lowest=7). The survey contained questions regarding participant characteristics (pain frequency, disease activity, impact on quality of life, experience, efficacy of treatments, healthcare role), treatment priorities they want to see in future research between all RCT interventions and treatment success definition priorities between all the ones used in the RCTs. Free text information could be added, where participants could add their own experiences and priorities (online supplemental files 5; 6).

In phase 2, results were collated, descriptively analysed and presented to the participants for further comment via email (online supplemental file 4). When comments were addressed, a final first draft of the priorities document was sent to the participants for review and the top three common research priorities for patients and healthcare professionals and research outcome measures from most important to least important were finalised.

Phase 3 aimed at triangulation of the phase 2 results and confirmation of saturation of the data via semi-structured group interviews, which participants from phase 2 were invited to attend online. Group instead of individual interviews were chosen to allow for the exchange of ideas. The interviews were organised over a 6-week period, each lasting approximately 1 hour. They were facilitated by MG with co-facilitation by other team members: VS and CG-C. Facilitators acted to ensure ground rules, confidentiality and to avoid the use of professional jargon. The semi-structured framework consisted of initial guiding questions, followed by open questions to encourage detailed answers and free expression of participant thoughts and ideas on the topics presented. Disagreements were managed via facilitation and discussion. Topics were based on the key consensus results from the Delphi process. These were presented to the participants in lay language during a short brief at the beginning of each session.

Consenting participants were allocated between four online interviews, with a plan to continue with more if saturation of data had not been achieved. The results of the Delphi process were discussed, and the aim was to explore areas of convergence and divergence. The interviews were held via Zoom and audio recorded. These were all manually transcribed into Word documents by RM, MA and NT, with pseudonyms for anonymity, that is, PT1, HP1. No record was kept regarding the participants names and their pseudonyms, to ensure the information cannot be tracked back to the participants. Once the transcriptions were completed, the audio recordings were destroyed.

CCUK were involved with the survey planning and involved in planning and recruiting for the Delphi workshops.

### Data analysis

The online survey results were analysed descriptively as numbers and percentages of participants and the average Likert scale, and are presented in tables. Missing data were not computed and results were calculated based on completed responses received per item.

Thematic analysis with inductive reasoning within a post-positivist framework was completed using one Excel spreadsheet by two independent researchers (RM and MA).^10^ The first thematic indices were further described, and novel themes were added based on the data extracted from the interviews, to ensure theoretical saturation was reached. The analysis followed three stages: open, axial and selective coding. Each stage provided categories that could be used to explore the themes of the data.

Reflexivity was practiced by the research team throughout the research process by journalling and memoing. This occurred in regular team meetings through all phases of the study and during the final analysis and supported reflecting on preconceived beliefs and assumptions and discussing these different thoughts among the team.^11^

A schematic model of the interactions between the factors that influence the choice of priorities, as identified above, was prepared by the authors.

## Results

### Online survey results (phase 1)

The survey was at least partially completed by 73 patients, 3 carers and 53 healthcare professionals (online supplemental tables 1,2).

For patients and carers, the highest-ranked research priorities were low FODMAP diet, cannabis and acupuncture (n=26/73, 36.1%). Healthcare professionals chose a low FODMAP diet as their number one research priority (n=29/52, 56.9%, table 1).

**Table 1:** Phase 1 survey results for treatment research priorities in inflammatory bowel disease-associated pain for patients and healthcare professionals, between all interventions that have been or are currently being tested in randomised controlled trials

	Healthcare professionals		Patients and carers		Total responses
Treatment	Research priority, n (%)	Rank	Research priority, n (%)	Rank	
Low FODMAP	29 (56.9)	1	26 (36.1)	1	55
Stress management course	28 (54.9)	2	19 (26.4)	5	47
Mindfulness	18 (35.3)	3	14 (19.4)	8	32
Online education	17 (33.3)	4	12 (16.7)	9	29
Relaxation therapy	13 (25.5)	5	20 (27.8)	4	33
Olorinab	11 (21.6)	6	15 (20.8)	6	26
Cannabis	10 (19.6)	7	26 (36.1)	1	36
Enteric-released GTN	10 (19.6)	7	15 (20.8)	6	25
Acupuncture	10 (19.6)	7	26 (36.1)	1	36
Yoga	6 (11.8)	10	7 (9.7)	12	13
Kefir diet	6 (11.8)	10	12 (16.7)	9	18
Stellate ganglion block	2 (3.9)	12	7 (9.7)	12	9
Transcranial DC stimulation	2 (3.9)	12	8 (11.1)	11	10
Daikenchuto	0 (0)	14	3 (4.2)	14	3

Rankings are relative to other treatments. Colour grading signifies priority position from highest (greenest) to lowest (reddest).

DC, direct current; FODMAP, fermentable oligosaccharides, disaccharides, monosaccharides and polyols; GTN, glyceryl trinitrate.

Patients ranked relaxation therapy (n=20/73, 27.8%) as their second research goal, followed by stress management courses (n=19/73, 26.4%). The second highest research goal for healthcare professionals were stress management courses (n=28/52, 54.9%), mindfulness techniques (n=18/52, 35.3%) and online education (n=17/52, 33.3%).

Olorinab, an agonist of cannabinoid receptor 2,^12^ was the 6th research priority for patients (n=15/73, 20.8%) and healthcare professionals (n=11/52, 21.6%). Enteric-released glyceryl trinitrate, a formulation that produces nitric oxide,^13^ ranked 7th for healthcare professionals (n=10/52, 19.6%) and 6th for patients (n=15/73, 20.8%).

The remaining items were ranked lower by both patients and healthcare professionals: kefir diet, yoga, stellate ganglion block, transcranial direct current (DC) stimulation and Daikenchuto, a traditional Japanese herbal medicine.^14^ Yoga was the 10th priority for health professionals, (n=6/52, 11.8%) and the 12th priority for patients (n=7/73, 9.7%). Healthcare professionals chose the kefir diet in 10th place (n=6/52, 11.8%), while patients ranked it 9th (n=12/73, 16.7%). Both healthcare professionals (n=2/52, 3.9%) and patients (n=7/73, 9.7%) considered stellate ganglion block as their 12th priority. Transcranial DC stimulation was graded 12th as a treatment goal by healthcare professionals (n=2/52, 3.9%) and 11th by patients (n=8/73, 11.1%). None of the healthcare professionals chose Daikenchuto as a research priority, while 3/52 (4.2%) patients classified it as their last therapy goal.

Patients’ and carers’ top three choices for treatment success definitions were ‘having no pain at all’ (average ranking=2.47), ‘improvement in the intensity of pain’ (average ranking=3.77) and ‘fewer days in which pain is present’ (average ranking=3.78) (table 2). For professionals they were ‘fewer days in which pain is present’ (average ranking=3.3), ‘improvement in the frequency of pain’ (average ranking=3.5) and ‘having no pain at all’ (average ranking=3.7) (table 3).

**Table 2:** Patients’ and carers’ and healthcare professionals’ priorities for treatment success outcomes in phase 1

Definition of treatment success (1=most important, 7=least important)	Patients and carers							Average	Healthcare professionals							Average
	1	2	3	4	5	6	7		1	2	3	4	5	6	7	
Having no pain at all	41	0	2	3	4	1	9	**2.47**	18	1	2	1	4	2	12	**3.7**
Improvement in the intensity of pain	4	11	18	8	5	9	5	**3.77**	5	5	13	7	4	3	3	**3.5**
Fewer days in which pain is present	3	21	7	7	6	9	7	**3.78**	2	7	8	7	9	6	1	**3.9**
Improvement in the frequency of pain	4	9	12	13	7	9	6	**4.02**	5	5	13	7	4	3	3	**3.5**
Change in pain intensity from ‘severe’ pain to ‘moderate’ pain	3	10	10	8	7	11	11	**4.38**	2	3	2	6	7	10	10	**5.1**
Fewer days with moderate or severe pain	2	5	7	14	15	10	7	**4.55**	2	7	8	7	9	6	1	**3.9**
A reduction in pain by at least 30%	3	4	4	7	16	11	15	**5.03**	4	5	4	5	3	10	9	**4.6**

**Table 3:** Phase 2 collective ranking of treatment priorities for both patients and healthcare professionals

Phase 2	
**Treatment**	**Collective rank**
Low FODMAP	1
Stress management course	2
Relaxation therapy	3
Enteric-release GTN	4
Acupuncture	5
Cannabidiol	6
Mindfulness	7
Online education	8
Ororinab	9

GTN, glyceryl trinitrate.

More results from phase 1 can be found in online supplemental tables 1-8.

### Phase 2

68 of the 128 original respondents responded in phase 2. The top three common research priorities for patients and healthcare professionals were low FODMAP diet (first), stress management therapy (second) and relaxation therapy (third). The research outcome measures from most important to least important were improvement in pain intensity, having no pain at all, improvement in pain frequency, fewer days with pain (table 4 and online supplemental tables 9-12).

**Table 4:** Phase 2 collective ranking of treatment success outcomes for both patients and healthcare professionals

Phase 2					
		**1=most important; 4=least important**			
	**Collective rank**	**1**	**2**	**3**	**4**
Improvement in the intensity of pain	**1**	22	23	6	7
Having no pain at all	**2**	27	2	7	22
Improvement in the frequency of pain	**3**	4	20	21	13
Fewer days in which pain is present	**4**	5	13	24	16

### Semi-structured group interview results

The online group interviews were attended by 13 patients and 5 healthcare professionals in total.

At the open phase of coding, we identified 205 unique subthemes with a total of 391 coded items. At the axial phase 16 overall themes were identified. These themes were: characteristics of IBD pain, patient with IBD as individual, patient beliefs and experiences, disease activity influencing symptoms and pain therapy choice, accessibility barriers, patients seeking therapies privately, desired multidisciplinary team (MDT) discussions, pharmacological pain therapies, cannabis use, non-pharmacological pain therapies, diet approach, low FODMAP diet, psychological therapies, interactions between diet and psychological therapies, quality of life and functional outcomes and research goals.

### Characteristics of IBD pain

The theme of pain was identified in various dimensions which bore a significant impact on patients with IBD. Pain location was identified as a pivotal factor that was linked to influencing pain therapies and varied among CD and patients with UC which could introduce a unique set of challenges to control it.

UC and Crohn’s disease are very different. Pain in UC is usually probably visceral hypersensitivity, IBS type symptoms, […] so probably responds quite well to things like a low FODMAP diet. In Crohn’s it’s far more complex. (CP1)

### Patient with IBD as an individual

The patient with IBD was identified as a unique individual who faces different challenges that revolve around themes of insufficiently individualised investigations, symptom individualism and concerns about future quality of life (QoL) especially regarding disease progression. Patient with IBD also highlight the importance of linking the influence of pain treatment to the type of pain and the patient’s background.

…doctors look at the result. Oh well, your tests are okay so you can’t possibly be having all these problems and pain and symptoms, because your markers are not off. So you don’t tend to get believed […] they don’t listen to the patient what the patient is telling them is no individualism at all. You’re all grouped under the same umbrella. (PT2)

### Patient beliefs and experiences

Patients reported a variation in healthcare professional attitudes towards them when they tried to seek medical care regarding IBD-related pain. Patients, who had negative experiences with their health professionals when accessing care, felt frustrated about their health professionals’ attitudes toward them and made them feel abandoned which negatively impacted their confidence in seeking medical help:

*…* if I don’t feel I’m getting what I need […] I can be quite a bolshie patient. And I think that it shouldn’t be that way, but that that is the experience that a lot of people have that you, you’ve got to fight for what you need. (PT3)

### Disease activity influencing symptoms and pain therapy choice

Healthcare professionals stated that they guide their IBD treatments based on disease activity. They envisage three possible scenarios for patients with chronic pain: those with active disease, responding or not to IBD treatments and those with inactive disease. Clinicians reported they were comfortable with managing the first group but acknowledged that in the presence of disease activity, inflammation takes all the focus, while pain is addressed less.

(…) I suppose the (…) slightly different issue is in those people who do have active inflammatory bowel disease but do have significant pain. We tend to get obsessed with treating the inflammation, and less good at treating the pain. (CP1)

### IBD treatment accessibility barriers

The accessibility of treatment for patient with IBD is a multifaceted challenge influenced by several key themes and present with its own barriers. Those barriers come in the form of financial constraints, age-related factors, geographical disparities, the COVID-19 impact, the absence of conclusive evidence and issues related to seeking help independently.

I do physiotherapy every 4-6 weeks because that’s what’s available in my area and that’s what I could afford to pay for. (PT5)

We do have dietary services, but they have a hell of a waiting list. (CP5)

### Patients seeking therapies privately

Patients with IBD might opt for seeking private care to get adequate medical care that is not provided by the National Health Service (NHS) like accessing a nutritionist or dietitian.

Personally, from my experience, I’ve been pretty much left to my own devices and, I seek out my own therapies,. (PT5)

Private nutritionist […] it costs but gives me good advice then measure nutrition levels vitamins, minerals etc […] I do think it makes a huge difference, but it isn’t something that’s ever really been mentioned through the NHS. (PT3)

### Desirable MDT discussions

Effective MDT discussions are crucial in dealing with the complex healthcare issues in patients with IBD.

You know, it’s a lot about talking to other people, collaborating to try to get the best care for the patients. (CP3)

### Pharmacological pain therapies

Patients with IBD experiencing pain stated that they have very limited analgesia options due to drugs being contraindicated in IBD or due to side effects.

we’re not allowed to take nonsteroidal anti-inflammatories, which, you know, do work very well for people who don’t have inflammatory conditions. We are limited, basically, to paracetamol, which kind of doesn’t work very well. Or coating with all sorts of side effects with it and then the other morphine based [drugs]. (PT4)

Tricyclic antidepressants are the pharmacological therapy that was discussed the most during the interviews. The impression was that there is a lack of evidence of their efficiency in clinical trials and there is more experience of using them in American hospitals, as opposed to the UK.

In my experience, physicians are uncomfortable using tricyclics, (…) [as] of the 6 tricyclics that are available in the United States, one is not the same as the other, you have to try different ones, you have to make sure you are not getting side effects particularly the weight gain, (…) so it takes a lot of work and effort I think to use tricyclics as chronic pain. (CP5)

### Cannabis use

Although patients have expressed interest in using cannabis for chronic pain in IBD, none of them had any experience of it in our interviews. Healthcare professionals reported a rise in being asked to prescribe it in clinics, but there is a lack of evidence and a legal framework.

(…) the pressure now on us now is that sometimes patients demand being prescribed cannabis and you just legally can’t, and also there is no evidence that it helps, and also CBD oil, which mechanistically shouldn’t do very much gets really hyped by patients. (CP4)

### Non-pharmacological pain therapies

Patients discussed about multiple non-pharmacological pain therapies, including hydrotherapy, acupuncture, physiotherapy, thermotherapy, with various levels of exposure and results.

(…) you try and deal with it yourself. Mind [you] if I can get to the gym and get in the pool, I am a happy bunny, because it helps me with my joint pains and my abdominal pain. (PT1)

(…) it kind of gives you back some control as well and it’s a non-medical intervention that I think we may be feeling a lot happier about it there’s no kind of side effects or limited side effects involved with it (…)*.* (PT3)

### Low FODMAP diet

Healthcare professionals reported they would use the FODMAP diet in patients with inactive IBD displaying symptoms of IBS, like early satiety, diarrhoea or bloating.

My patients who have persistent symptoms that sound as if they would otherwise be of irritable bowel type spectrum, and I am quite happy that their disease is well controlled, [low FODMAP diet] would be the first line therapy. (CP3)

Patients emphasised that a low FODMAP diet has limited availability in the NHS and can be quite restrictive and laborious, but it had the biggest impact on their pain compared with other non-pharmacological therapies.

FODMAP diet if I am careful, actually does give me more control than anything else. But at the time, 20 odd years ago, I had to actually push to do it. But it did have a big effect. (PT5)

### Psychological therapies

Patients prefer psychological therapies as this helps them to cope with having a chronic illness and with everyday pain. Most patients experimented with yoga, relaxation therapy, stress management techniques, physiotherapy and meditation, mostly in the private sector.

[Mediation] gives me a calmer way to start the day and close the day I suppose it kind of resets so it’s kind of waking up and hitting the day with your head fizzing it’s like it’s a mental reboot you just kind of have some calm time. (PT7)

In theory, [hypnotherapy and cognitive behavioural therapy] are good targets but realistically unless you have access to a very specialized unit, which just happens to have a sort of a psychologist attached. I would think we’re talking in the units in one hand probably they have that available. (CP2)

### Interactions between diet and psychological therapies

Patients have repeatedly reported that there is a strong connection between their mental state and their dietary habits.

…my dietary habits and stress but also [other] patients [said] that the more stressed they are, the less they pay attention to their diet, all goes out of the window. (PT6)

If you drive [disease activity] down you drive down the symptoms and you can equally drive down the anxiety because basically the things that people get anxious about are less likely to happen because they know they can get a control back. (CP2)

### Quality of life and functional outcomes

The new theme that was identified during the interviews is functionality as a research goal.

[I would aim for] improvement to make pain manageable so you can get on with your life, even though you’ve got chronic pain*.* (PT8)

Patients repeatedly expressed that being pain-free would allow them to be active, and implicitly their quality of life would improve.

Can I get up and get dressed and go out and do things or do I need to stay in bed. Go to work. Or am I stuck at home because I need to be wired this way now. Can I go and see my friends or my family now that lockdown’s over or am I in too much pain that is very much. (PT9)

### Research priorities

Patients and healthcare professionals reported that having ‘no pain’ as a research goal would be unrealistic, and it would render all therapies as inefficient.

it’s a bit idealistic to imagine that maybe pain would never, ever be there, even though we would all like that, but if you could make it a bit easier, it would help. (PT9)

Most participants from both groups opted for reducing the intensity of acute pain and the frequency of chronic pain as a research priority.

…for acute pain, I would agree with that, improvement in the intensity of pain, that should be number one. For chronic pain, my experience (…) is that intensity is not the problem, intensity is the same all the time. (…) So, with chronic pain, I would put improvement in the frequency of the pain. (CP5)

### Proposed interaction model for the factors underpinning patient and professional choices for future research goals

**Figure 1:**
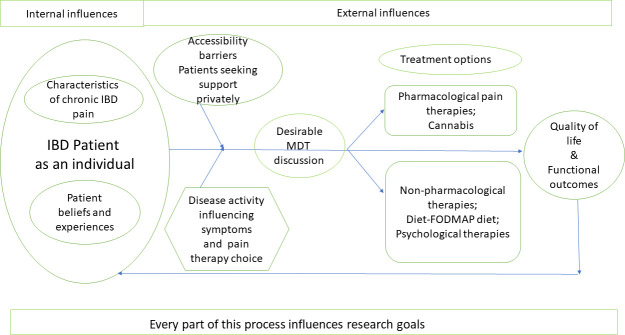
Schematic model of the interaction between internal and external factors influencing patient and healthcare professional research priority choices for the treatment of IBD-associated pain. FODMAP, fermentable oligosaccharides, disaccharides, monosaccharides and polyols; IBD, inflammatory bowel disease; MDT, multidisciplinary team.

At the core of all themes lies the patient with IBD as an individual, alongside characteristics of IBD pain and personal beliefs and experiences. Patients access pharmacological or non-pharmacological pain therapies, as dictated by a multitude of factors: MDT approach, accessibility barriers, disease activity and patients seeking treatment privately. The outcome of pain therapies impacts the quality of life, functional outcomes and implicitly the starting point: patients with IBD, their pain characteristics, beliefs and experiences. Research goals were proposed by patients and healthcare professionals, as a result of the interaction of all these internal and external factors (figure 1).

Online supplemental table 13 provides a summary of the main themes and quotes followed by additional quotes.

## Discussion

Our study is a co-production of views on chronic pain in IBD and to our knowledge this is the first study to engage with patients on such a key topic.

Our study identified low FODMAP diet and psychological therapies as the top research priorities for patients and healthcare professionals in chronic pain in IBD. Patients also raised concerns that their pain symptoms are not addressed appropriately by clinicians, that pain therapies are not available in the NHS, which affects their quality of life, functionality and pushes them to seek advice privately. We believe this patient co-produced study is vital in determining the next research and clinical priorities for the wider IBD community.

By identifying research priorities in chronic pain in IBD, we hope that future studies will focus on these goals and the quality, as well as certainty, of evidence will improve. It is vital to explicitly state that such research may be just as likely to discount therapies based on their efficacy or safety, as it would be to support their use. However, by targeting research efforts in these core areas, the speed at which this point in the evidence base and onward clinical guidance can be reached. Such an approach where evidence synthesis clearly and precisely inform prioritisation for future research in turn will enhance the certainty of findings in future synthesis. Even though this appears self-evident such reflexive use of synthesis findings appears very rare in this field, like many others. Linking the two phases with the novel approach of the study which included patients at the centre of such prioritisation could set a model for other researchers. By prioritising our participants’ research goals in future studies, we will be able to reach a good level of certainty in the next Cochrane reviews for these priority treatments. Furthermore, our study could help prioritise other interventions for chronic pain that we did not mention.

We suggest to international societies and national bodies to consider and endorse our prioritisation findings in promoting cost-effective research funding. Researchers could cite our article as justification for funding applications. Our novel interaction model reflects the internal and external influences on patients and health professionals when choosing the chronic pain in IBD research goals. The model identifies the central role of patients’ voice in leading the research prioritisation required. There were areas of disagreement between participants. For example, while health professionals found the goal of having no pain at all unrealistic, some patients believed that we should still work towards this goal. Otherwise, this will impact on long-term hope and patients will have to resign to the thought that their pain will never be resolved.

The limitations of our study include our data analysis method, which is open to interpretation bias on the part of the researchers, with our own preconceived ideas shaping the analysis. Every effort has been made to minimise such bias, by having two independent researchers in formulating the open and axial themes. If there was disagreement, the two researchers would discuss these and reach a consensus, in line with best practice. Another possible source of bias is that the study is based on a volunteer sample. Although it covers a wide range of geographical regions, genders and ages, it is possible that the participants may have more severe disease or chronic pain, which has made them interested in research, but their interests may not be representative of the wider population. Patients had very different exposures to treatments of chronic pain depending on local availability or personal experience and this also had an impact on their responses. Social acceptability bias is also possible, with respondents censoring opinions they felt would be unacceptable. Given these limitations, further study is needed to confirm the features of our proposed model and, in particular, the applicability of our findings in daily clinical practice and research.

Future research is implicit within our research goals and findings regarding pain treatments. However, our novel conceptual model may have implications more broadly in other research prioritisation in IBD and we would invite other researchers to consider this approach. We would also encourage such large sampled and multistage co-production is considered more broadly within research prioritisation in IBD to ensure the patient voice is recognised and actioned.

## Conclusions

Low FODMAP diet, followed by psychological therapies were the highest rated research priorities for healthcare professionals and patients. We would recommend funding bodies and researchers to consider this, as well as our proposed conceptual model for understanding these findings, when making choices for future research, as well as to guide future prioritisation exercises.

## Supplementary material

10.1136/bmjgast-2024-001483 online supplemental file 1

10.1136/bmjgast-2024-001483 online supplemental file 2

10.1136/bmjgast-2024-001483 online supplemental file 3

10.1136/bmjgast-2024-001483 online supplemental file 4

10.1136/bmjgast-2024-001483 online supplemental file 5

10.1136/bmjgast-2024-001483 online supplemental file 6

## Data Availability

Data are available upon reasonable request.
