# Supplementary material for: Patients’ and health professionals’ research priorities for chronic pain associated with inflammatory bowel disease: a co-produced sequential mixed methods Delphi consensus study
Source: BMJ Open Gastroenterol. 2024 Sep 12;11(1):e001483. doi: 10.1136/bmjgast-2024-001483 (PMC11404265; doi:10.1136/bmjgast-2024-001483)
Supplement: online supplemental file 1 [file bmjgast-11-1-s001.pdf]

	Crohn’s disease	Ulcerative colitis	Overall, n (%)
**Number of responses**	51	25	76 (100)
**Respondent type**			
Patient	49	24	73 (96.1)
Carer	2	1	3 (3.9)
**Age group**			
Adult	51	24	75 (98.7)
Paediatric	0	1	1 (1.3)
**Length of disease**			
< 1 year	1	1	2 (2.7)
1 - 3 years	8	3	11 (15.1)
3 - 5 years	6	2	8 (11.0)
5 - 10 years	4	4	8 (11.0)
> 10 years	30	14	44 (60.3)
**Remission**			
Yes	27	13	40 (54.8)
No	22	11	33 (45.2)
**Pain frequency**			
At least once a day	13	6	19 (26.0)
A few times a week	16	5	21 (28.8)
A few times a month	11	2	13 (17.8)
A few times a year	6	6	12 (16.4)
No pain in the last 12 months	3	4	7 (9.6)
**Impact on QoL**			
1 (insignificant)	8	5	12 (16.4)
2 (minor)	9	7	16 (21.9)
3 (moderate)	16	6	22 (30.1)
4 (major)	10	1	11(15.1)
5 (in a significant way)	8	5	13 (17.8)

Supplementary table 1. Characteristics of patients and carers who participated in Phase 1.

**Healthcare role (n=52)**	**n (%)**
Doctor (consultant)	31 (59.6)
Doctor (trainee)	8 (15.4)
General practitioner	5 (9.6)
Specialist nurse	5 (9.6)
Other	3 (5.8)
**How frequently do patients ask for support in managing IBD-associated pain?**	**n (%)**
Never	0 (0.0)
Infrequently	8 (15.7)
Sometimes	26 (51.0)
Often	14 (27.5)
All the time	3 (5.9)

Supplementary table 2. Healthcare roles of healthcare professionals who participated in phase 1 and how often they're asked for support in managing IBD-associated pain

Treatment	Aware of treatment, n (%)	Used treatment, n (%)	Has been recommended this treatment by a healthcare professional, n (%)	Not aware, used or recommended, n (%)
Low FODMAP	38 (50)	12 (15.7)	5 (6.5)	18 (23.6)
Acupuncture	18 (23.6)	8 (10.5)	0 (0)	47 (61.8)
Mindfulness	24 (31.6)	15 (19.7)	2 (2.6)	32 (42.1)
Stress management course	24 (31.6)	15 (19.7)	1 (1.3)	33 (43.4)
Enteric-released GTN	1 (1.3)	0 (0)	0 (0)	72 (94.7)
Olorinab	0 (0)	0 (0)	0 (0)	73 (96)
Online education	12 (15.7)	6 (7.9)	1 (1.3)	54 (71)
Relaxation therapy	16 (21)	14 (18.4)	1 (1.3)	42 (55.2)
Yoga	19 (25)	13 (17.1)	0 (0)	41 (53.9)
Transcranial DC stimulation	0 (0)	0 (0)	0 (0)	73 (96)
Kefir diet	26 (34.2)	4 (5.2)	0 (0)	43 (56.5)
Stellate ganglion block	0 (0)	0 (0)	0 (0)	73 (96)
Daikenchuto	1 (1.3)	0 (0)	0 (0)	72 (94.7)
Cannabidiol	2 (2.6)	7 (9.2)	0 (0)	64 (84.2)

Supplementary table 3. Phase 1 survey results for patients'/carers' experience with treatment for IBD pain, for interventions that have been or are currently being tested in randomised controlled trials.

Treatment	Aware of treatment, n (%)	Has recommended this treatment, n (%)	Not aware or recommended, n (%)
Low FODMAP	16 (31.4)	21 (41.2)	14 (27.5)
Acupuncture	32 (62.7)	7 (13.7)	12 (23.5)
Mindfulness	27 (52.9)	14 (27.5)	10 (19.6)
Stress management course	23 (44.2)	13 (25.0)	16 (30.8)
Enteric-released GTN	4 (7.8)	1 (2.0)	46 (90.2)
Olorinab	5 (9.8)	1 (2.0)	45 (88.2)
Online education	19 (37.3)	17 (33.3)	15 (29.4)
Relaxation therapy	21 (41.2)	5 (9.8)	25 (49.0)
Yoga	23 (45.1)	7 (13.7)	21 (41.2)
Transcranial DC stimulation	11 (21.6)	1 (2.0)	39 (76.5)
Kefir diet	12 (23.5)	7 (13.7)	32 (62.7)
Stellate ganglion block	9 (17.6)	2 (3.9)	40 (78.4)
Daikenchuto	1 (2.0)	1 (2.0)	49 (96.1)
Cannabidiol	35 (68.6)	5 (9.8)	11 (21.6)

Supplementary table 4. Phase 1 survey results for healthcare professionals' experiences with treatments for IBD-associated pain, for interventions that have been or are currently being tested in randomised controlled trials.

	1 = no effect; 5 = maximum effect					
	**1**	**2**	**3**	**4**	**5**	**Average**
Mindfulness	1	7	3	3	1	2.7
Cannabidiol	0	3	4	0	0	2.6
Acupuncture	2	2	2	2	0	2.5
Relaxation therapy	4	4	4	3	0	2.4
Low FODMAP	3	4	3	2	0	2.3
Stress management course	2	4	3	1	0	2.3
Yoga	4	4	5	0	0	2.1
Kefir diet	1	2	1	0	0	2
Online education	4	1	0	1	0	1.7
Stellate ganglion block	0	0	0	0	0	0
Daikenchuto	0	0	0	0	0	0
Enteric-released GTN	0	0	0	0	0	0
Olorinab	0	0	0	0	0	0
Transcranial DC stimulation	0	0	0	0	0	0

Supplementary table 5. Phase 1 survey results for perceived efficacy of treatments patients have used for IBD-associated pain, for interventions that have been or are currently being tested in randomised controlled trials.

	Respondents	1 = no effect; 5 = maximum effect					
		1	2	3	4	5	Average
Daikenchuto	1	0	0	0	0	1	5.0
Acupuncture	7	0	0	4	1	0	4.0
Mindfulness	14	0	1	6	4	0	3.9
Relaxation therapy	17	0	6	4	6	0	3.6
Online education	5	0	1	2	1	0	3.6
Cannabidiol	5	0	1	0	4	0	3.6
Yoga	7	0	3	2	0	0	3.4
Low FODMAP	31	0	4	14	13	0	3.3
Stress management course	13	0	5	3	2	2	3.2
Stellate ganglion block	2	0	1	0	1	0	3.0
Enteric-released GTN	1	0	0	1	0	0	3.0
Olorinab	1	0	0	1	0	0	3.0
Kefir diet	7	0	5	2	0	0	2.3
Transcranial DC stimulation	1	0	1	0	0	0	2.0

Supplementary table 6. Phase 1 survey results for perceived efficacy of treatments healthcare professionals have used for IBD-associated pain, for interventions that have been or are currently being tested in randomised controlled trials.

Other treatments used by patients for IBD pain		
Codeine (n = 6)	Paracetamol (n = 5)	Co-codamol (n = 1)
Hot water bottle (n = 5)	Pain pod (n = 1)	Buscopan (n = 4)
Mebeverine (n = 1)	Windeze (n = 1)	Peppermint tea (n = 1)
Laxido (n = 1)	Infliximab (n = 1)	Humira (n = 1)
Azathioprine (n = 1)	TPN enteral diet (n = 1)	Walking (n = 1)
Steroids (n = 2)	Marijuana (n = 2)	Oxycodone (n = 2)
Tramadol (n = 4)	Dihydrocodeine (n = 1)	Morphine (n = 1)
Opioids (n = 4)	Herbal remedies (n = 1)	Liquid diet (Modulen IBD) (n = 1)
Vitamin D (n = 1)	Probiotics (n = 1)	Mint drops (n = 1)
Wormwood brew (n = 1)	Imodium (n = 1)	Amitriptyline / nortriptyline (n = 2)

Supplementary table 7. Other treatments used by patients for IBD-associated pain from phase 1 survey results.

Other treatments recommended by healthcare professionals for IBD pain		
Gabapentin (n = 2)	Hypnosis (n = 1)	Pain modulating therapy, such as imipramine (n = 1)
Standard analgesics	Low dose tricyclic antidepressants (n = 2)	Paracetamol (n = 1)
Pregabalin (n = 1)	Peppermint (n = 1)	Anti-spasmodics (n = 1)
Low-dose naltrexone (n = 1)	Probiotics (n = 1)	Low residue diet (n = 1)
Laxatives (n = 1)	Amitriptyline	Gabapentinoids (n = 1)
Codeine (n = 1)	Polymeric diet (n = 1)	Neuropathic pain medications (n = 1)

Supplementary table 8. Other treatments recommended by healthcare professionals for IBD-associated pain from phase 1 survey results.
