# Supplementary material for: Patients’ and health professionals’ research priorities for chronic pain associated with inflammatory bowel disease: a co-produced sequential mixed methods Delphi consensus study
Source: BMJ Open Gastroenterol. 2024 Sep 12;11(1):e001483. doi: 10.1136/bmjgast-2024-001483 (PMC11404265; doi:10.1136/bmjgast-2024-001483)
Supplement: online supplemental file 2 [file bmjgast-11-1-s002.pdf]

	Phase 1				Phase 2	
Treatment	HCP Rank	Difference	Patient / Carer Rank		Treatment	Collective rank
Low FODMAP	1	0	=1		Low FODMAP	1
Stress management course	2	3	5		Stress management course	2
Mindfulness	3	5	8		Relaxation therapy	3
Online education	4	5	=9		Enteric-release GTN	4
Relaxation therapy	5	1	4		Acupuncture	5
Ororinab	6	0	=6		Cannabidiol	6
Cannabidiol	=7	6	=1		Mindfulness	7
Enteric-released GTN	=7	1	=6		Online education	8
Acupuncture	=7	5	=1		Ororinab	9
Yoga	=10	2	=12			
Kefir diet	=10	1	=9			
Stellate ganglion block	=12	0	=12			
Transcranial DC stimulation	=12	1	11			
Daikenchuto	14	0	14			

Supplementary table 9. Treatment priorities rankings from phases 1 and 2

Low FODMAP	Stress management course	Relaxation therapy	Enteric-release GTN	Acupuncture	Cannabidiol	Mindfulness	Online education	Ororinab
Patients like dietary approaches and may have features which overlap with IBS<br><br>Diet seems to cause symptoms.<br><br>I have heard of this diet. I would like to base by diet decisions on research.<br><br>I am unsure of the associated benefits of food.<br><br>I believe it works for some people, it didn't make any difference to me.<br><br>Should be a low carbohydrate diet for more effect.<br><br>Diet before medication.<br><br>Diet major contributor to my illness.<br><br>I think that dietary approaches are key.<br><br>Diet in general has a big role in IBD pathophysiology.<br><br>It has made a big difference for me, but I had to push to try nutritional management.<br><br>Best evidence for effectiveness.<br><br>Helps in some but not others.<br><br>Seems to be a lot of talk about its benefits.<br><br>It's cost-effective for the patient.<br><br>Build on a body of evidence that already shows positive outcomes in IBS.<br><br>Not enough knowledge on what food actually helps.<br><br>Already available. Safe. Would be relatively easy to implement and use.<br><br>Not enough priority given to food management especially during times of pain.<br><br>The NHS does not focus on diet	Stress brings on Crohn's disease<br><br>Pain has a large psychological profile, but you don;t tell us which affective disorder the Crohn's patient has. This makes selecting psychological treatment more difficult.<br><br>Better able to manage life with reduced impact of the disease and also improve general well being.<br><br>My stress levels made me feel worse.<br><br>Suffering with IBD brings with it anxiety, being able to deal with this without prescription would be helpful.<br><br>Stress is a big part of flare up.<br><br>Stress makes pain and condition worse.<br><br>The link between stress and the onset / flaring of IBD could benefit many people.<br><br>Due to the link between stress and gut health.<br><br>Stress does make things worse and also causes IBS symptoms.<br><br>As stress can cause so many health issues.<br><br>Stress is a key gator for me.<br><br>Living with the condition can make stress worse which then makes the condition worse.<br><br>Stress plays a part in how pain affects people.<br><br>Stress is there whether acknowledged or not.<br><br>Patients with pain have a high psychological burden that should be addressed.<br><br>Stress is a killer.<br><br>More about the mind and non-invasive.<br><br>Such courses involve high resource and studies may increase access to resources to patients sooner, and demonstrate the value in investing in them.	To help cope with illness.<br><br>More useful for anxiety than depression.<br><br>Any treatment is worth trying for a chronic disease.<br><br>To be able to relax and not worry about toilet mapping or worrying about mess.<br><br>It works.<br><br>Being stress free actually helps with IBD pain so I feel there should be more research into this especially for newly diagnosed patients.<br><br>Helps with stress.<br><br>Potential to have beneficial effects beyond pain in IBD.<br><br>Stress is a big trigger for the pain.<br><br>Relaxation may lessen pain.<br><br>Same with mindfulness, can be done anywhere and anytime once you get the hang of it.<br><br>Patients with pain have a high psychological burden that should be addressed.<br><br>Help to reduce stress and anxiety.<br><br>Such therapy involves high resources and studies may increase access to resources for patients sooner, and demonstrate the value in investing in them.<br><br>As with mindfulness, this is key because living with a painful, debilitating long-term condition can be very stressful. The pace of life these days is often frantic and can contribute to stress, which is known to be an exacerbating factor for Crohn's flare ups.	Not sure what this is would like to know more.<br><br>Patient request.<br><br>Interesting effects on healing of MSK injury so would be curious to know if it has any effect.<br><br>Relief during flare ups.<br><br>Potential for benefit.<br><br>Unsure.<br><br>Something I've seen isn't as effective in Crohn's disease.<br><br>Seems likely to be safe and easy-to-use.<br><br>Less invasive treatment than many currently available.<br><br>Is this effective and how does it work?<br><br>Something new worth trying?<br><br>The mechanism of action sounds like one worth exploring for pain relief in IBD, which could be an alternative to opioids.<br><br>Most likely to be utilised by medical professionals than CBD.<br><br>Interesting idea.<br><br>Need to have all medications possible.<br><br>It is a new concept to me and it would be good to know the scientific basis for its use and effectiveness.<br><br>Possible new treatment?<br><br>Does this work? I've read mixed reviews.	I have been having acupuncture from someone from the British acupuncture Council for some time now as my joints have been affected. I was offered hydrotherapy at the hospital, and so tried acupuncture instead as I felt totally crippled.<br><br>Understanding the possible benefits of acupuncture could lead to real treatment that wouldn't require taking medications.<br><br>It would be good to see if it actually works.<br><br>We know so little about this in Western medicine but it clearly is very effective in China.<br><br>Something I want to try as I've heard it does help with IBD symptoms but when I tried to research it is not enough research.<br><br>Appears popular with patients in my experience.<br><br>May relieve pain and help.<br><br>Traditional Chinese medicine.<br><br>I've heard good things about it.<br><br>Physical and psychological response.<br><br>It can be very successful for pain.<br><br>I have found acupuncture to be useful for injuries, worth a try?<br><br>It's potential to balance the body and alleviate stress stop	Patient request.<br><br>Might be worth a shout, or is it just all in the news at the moment?<br><br>Proven relief for pain.<br><br>Joint pain associated.<br><br>I have heart cannabidiol helps in some cases, but not sure how.<br><br>Given a bad rep but can be very helpful.<br><br>Cannabis is super food.<br><br>Lots of people seem to report good results.<br><br>Interesting new treatment, might be less invasive than steroids/biologics.<br><br>Does this safely have an effect on pain?<br><br>Easily accessible on the high street.<br><br>Possible natural pain relief with less toxic side-effects.<br><br>Trying a more natural medication.<br><br>This really needs looking into.<br><br>Treatment that is natural and may relieve pain and not have lasting effects is a must to me.<br><br>It has become very popular in 'health food' shops lately and many people might be tempted to sell Medicaid. It would be good to know the scientific basis for its use.<br><br>Some of the medicines on offer have horrific side effects. Would support being able to use something more natural.<br><br>Does this really work and should it be recommended?	Better for younger patients with mostly anxiety.<br><br>Any relaxation. Does it help the body heal?<br><br>The link between the brain and IBD control could be life changing for many.<br><br>This does help people to manage symptoms better.<br><br>Most of the people I know with IBD are busy achievers, not very good at switching off!<br><br>Can be guided or self-management, can be done anytime and anywhere.<br><br>Patients with pain have a high psychological burden that should be addressed.<br><br>Non-invasive.<br><br>Very important.<br><br>Absolutely key to managing a long-term condition. I find this immensely helpful myself and stopped negative thoughts spiralling out of control and makes you more aware of your own body and any tensions and part of it which may be contributing to symptoms. A better mind-body relationship will help with the more holistic, personal aspects of managing the illness for each individual.	To learn how to deal with illness<br><br>As patients use online sources increasingly known how to do it well and any SFX would be a wise idea.<br><br>Knowledge about treatments available would be useful.<br><br>Understanding what is happening to your body can help you understand and see things in perspective.<br><br>Education for patients.<br><br>Available to patients and support around them.<br><br>Better education leads to less stigma and fear.<br><br>It is vital for people to know the facts.<br><br>Help those who cannot get out and to be able to do their own research.<br><br>To educate new patients.	Seems to have merit.<br><br>Patient request.<br><br>Supposed to be a good pain relief.<br><br>Gut specific pain.<br><br>If we can find effective pain relief, it will reduce stress linked with pain as a really good starting point. Buscopan is the only drug that helps my spasms and it takes a good while to kick in. Not easy when I'm working.<br><br>I don't know about it!<br><br>Haven't heard about it before.<br><br>The most novel of the approaches. Potentially the greatest gain. Simple treatment if shown to work.<br><br>This sounds like a promising alternative to opiates for long-term pain management in IBD.<br><br>More likely to be utilised by medical professionals than CBD.<br><br>Important.<br><br>Interesting research.<br><br>New treatments should always be a priority so we have a better choice.<br><br>It is a new concept to me and it would be good to know the scientific basis for its use and effectiveness.

very much.<br><br>Diet is important so any information what is good or to be avoided is useful.<br><br>Lifestyle change. Can be guided by dietician or self management.<br><br>This is a relatively safe treatment option.<br><br>Not only FODMAP but vegetarian.<br><br>More advice on what to change in diet.<br><br>Diet plays a big part of managing Crohn's disease and some foods classed as easily digestible actually are not and hospital nutritionists need to realise this.<br><br>To exclude food intolerance.<br><br>Interesting to see results of further studies of this intervention used often in clinical practice.<br><br>I think diet is absolutely fundamental to manage Crohn's disease and I have personally obtained relief through a Gluten-free diet which incidentally is low in FODMAPs. It is also well known to help people with IBS so I think it could help with issues that Crohn's sufferers face too such as abdominal cramps and bloating.<br><br>Die aspect of IBD is not sufficiently researched.<br><br>Because some of the recommended foods make me worse. I'd like to know what really works.								

Supplementary table 10. Comments on treatments as collected in Phase 2.

	Phase 1				
	Professional			Patient	
	Average	Rank	Difference	Rank	Average
Fewer days in which pain is present	3.3	1	2	3	3.78
Improvement in the frequency of pain	3.5	2	2	4	4.02
Having no pain at all	3.7	3	3	1	2.47
Fewer days with moderate or severe pain	3.9	4	4	6	4.55
Improvement in the intensity of pain	4	5	5	2	3.77
A reduction in pain by at least 30%	4.6	6	6	7	5.03
Change in pain intensity from 'severe' pain to 'moderate' pain	5.1	7	7	5	4.38

	Phase 2				
		1 = most important; 4 = least important			
	Rank	1	2	3	4
Improvement in the intensity of pain	1	22	23	6	7
Having no pain at all	2	27	2	7	22
Improvement in the frequency of pain	3	4	20	21	13
Fewer days in which pain is present	4	5	13	24	16

Supplementary table 11. Treatment outcome rankings from phases 1 and 2

Improvement in the intensity of pain	Having no pain at all	Improvement in the frequency of pain	Fewer days in which pain is present
Depends on how much is due to inflammation how much to over reporting from anxiety/depression. If over reporting, this won't change much.<br><br>Less pain is better.<br><br>I feel this will be life transforming as a patient. Some pain I can live with, have some normalcy in my day-to-day life.<br><br>It would be beneficial to know why it is so intense and the causes etc.<br><br>If the pain is not as bad, it would be more tolerable.<br><br>So you can tolerate the pain.<br><br>Improvement in the intensity of pain would be a start.<br><br>Intense pain can be debilitating and make carrying on with normal life really hard. Reducing the intensity would be life changing.<br><br>Can function day-to-day with mild pain but becomes harder with increased intensity and impossible when doubled up.<br><br>Patient -related target.<br><br>Right like pain can be doubling over so would like to sit or stand without bending over to relieve.<br><br>Pain is debilitating and I've gained weight.<br><br>For me the intensity of the pain is something I struggled with as once I get really bad pain I get other symptoms too such as nausea and dizziness which is why it's important to me as this is something I struggled with the most.<br><br>Will help the patient.<br><br>The pain is unbearable.	This would be the perfect world!<br><br>Surely this is the ultimate objective?<br><br>Nobody wants pain.<br><br>I would wish to be pain-free.<br><br>So I can live like a normal person.<br><br>Not having pain could mean being able to live a normal life.<br><br>This is the best option to achieve.<br><br>The ultimate aim, but realistically may not be achievable for all.<br><br>Is this even possible?<br><br>Forgotten what this feels like.<br><br>This would be amazing if no pain was ever experienced.<br><br>Would likely require larger trials.<br><br>This would be ideal.<br><br>That is what we would want.<br><br>This would be the ultimate goal.<br><br>A very ambitious goal. Likely not often achieved.<br><br>More manageable.<br><br>Better pain control needed.<br><br>It would be life changing.<br><br>To be more normal.<br><br>Being pain-free is my goal.<br><br>Very important have no pain at all.<br><br>This is the 'holy grail' - what everyone wants.<br><br>Least likely to be a demonstrate will outcome in a trial,	Again, depends on how much is psychological versus inflammatory.<br><br>Less frequent is more bearable.<br><br>It would be nice to know how we can avoid pain.<br><br>The less amount of pain is better.<br><br>You will never be pain-free but reducing hours is less stressful.<br><br>Less pain means I can do more.<br><br>Less pain often means an improvement in life quality and productivity and 'normality'.<br><br>No particular reason.<br><br>To increase ability to socialise when food is involved, manage work with pain.<br><br>Better quality of life.<br><br>As it's always the same time in the morning which inhibits activities.<br><br>For me, I get the pain every day and it can come at any time. I would love for there to be something to stop how frequent the pain could come.<br><br>Will help the patient.<br><br>Perceived patients with value this more than improvement in intensity.<br><br>The pain I get is unbearable.<br><br>To have less pain.<br><br>This is another way of measuring the number of days, they are more or less equivalent outcomes.	Might have the best chance of success. No pain is probably unrealistic.<br><br>In this pain is wearing.<br><br>This will allow me as a patient to have some hope/expectation that pain would not be a constant in my life.<br><br>Bit of relief in between bad days.<br><br>Quality of life.<br><br>It's all a bit arbitrary and patients differ.<br><br>Quality of life.<br><br>Mentally give yourself a rest.<br><br>If not pain-free then fewer days with pain.<br><br>Less pain means you can do more.<br><br>Overall quality of life improvement.<br><br>Realistic and achievable target.<br><br>Most helpful to patients I think.<br><br>Fed up of everyday pain.<br><br>Less days of pain.<br><br>Pain-free days can increase productivity and well-being.<br><br>To catch a break from pain.<br><br>Frequency is easy to measure but patients seem less bothered by this than intensity.<br><br>To improve overall quality of life.

To reduce pain to bearable levels.<br><br>Intensity seems the most bothersome to patients, reducing this seems to have the most gains.<br><br>To have less pain would be an improvement.<br><br>The most difficult to manage.<br><br>I suspect pain will always be a feature of IBD so the intensity can be reduced that would make a big difference to many people.<br><br>Allowing patients to manage their life better.<br><br>Less intense is easier to carry on and live with.<br><br>Personally, improvement in pain intensity would be most beneficial to quality of life because the pain will be less debilitating, allowing one to undertake more regular activities.<br><br>More bearable pain intensity will help patients cope better.<br><br>Lesson the reliance on coping mechanisms.<br><br>Extreme pain affects how an individual mentally and emotionally copes with chronic illness.<br><br>Better pain control is needed.<br><br>Lesson the reliance on coping mechanisms.<br><br>Extreme pain affects how an individual with chronic disease copes mentally and emotionally.<br><br>Life would be easier.<br><br>If having no pain at all can't happen I'd like to not experience intense pain.<br><br>Reduce pain levels.<br><br>It is possible to live with low level pain but intense pain can be devastating.<br><br>So important because if pain reaches a certain level it can make it very difficult to lead a normal life, attend work/school and do the things you need and want to	but an important outcome to look at.<br><br>Imagine!!!<br><br>I could forget a chronic disease.<br><br>Probably unrealistic to expect this.	To have days when pain is not present would help our mental health.<br><br>To make days easier - juggling work, family, chores etc.<br><br>This would make a big difference to day-to-day life.<br><br>This will allow patients to have a better quality of daily life.<br><br>Less frequent can live with.<br><br>Improvement in pain frequency will improve quality of life by providing some respite from pain.<br><br>Less likely to occur in the day.<br><br>Lesson the number of 'rest' days needed.<br><br>Being able to decrease the frequency of pain would give feeling of more control.<br><br>Intense pain stops you doing so much.<br><br>Sleep and quality of life.<br><br>Frequent pain can be very draining and depressing.<br><br>Allow patients more functional time is most important.<br><br>Pain gnaws away at the edge of your consciousness the more intense it is the less you are able to do.<br><br>Less time spent in pain as good.	This would be a great outcome, for me it is probably harder to achieve than a general reduction in intensity and frequency.<br><br>Eventually learn to distract on the bad days.<br><br>Some have them for too long so a goal like this is more realistic, baby steps to eventually becoming pain-free.<br><br>As an IBD sufferer, I am resigned to having some pain, but few are days when experiencing pain would improve my quality of life.<br><br>Self-explanatory.<br><br>Better quality of life.<br><br>To allow more freedom to lead a more 'normal' lifestyle.<br><br>Quicker control of any level of pain would cause less upset and life disturbances.<br><br>Better pain control needed.<br><br>Make life more pleasant.<br><br>To be able to work better and socialise.<br><br>Quality-of-life.<br><br>Pain is debilitating for me and has a huge impact on my quality of life. I'd like to not miss out on things because of pain.<br><br>Need have pain-free days.<br><br>Allowing patients more functional time is most important.<br><br>This would be preferable in my opinion to having low intensity pain but having it every day. At least if you can have more 'good' days where you feel reasonably normal, you can be more productive in your daily life and feel less beaten down by the constant pain.<br><br>Again, less days of pain is a good thing.<br><br>Pain is so draining and this would make everything easier.

do.<br><br>Less crippling, can’t get out of bed.<br><br>Because pain is so tiring and debilitating.<br><br>You get used to lower levels of pain and it interferes less with your life.<br><br>Less impact.<br><br>Less pain when I have pain means able to cope better with it.			Gives days with less impact on day-to-day activities.<br><br>Better able to get on with life without pain stop

Supplementary table 12. Comments on treatment outcomes as collected in Phase 2.
