# Supplementary material for: Patients’ and health professionals’ research priorities for chronic pain associated with inflammatory bowel disease: a co-produced sequential mixed methods Delphi consensus study
Source: BMJ Open Gastroenterol. 2024 Sep 12;11(1):e001483. doi: 10.1136/bmjgast-2024-001483 (PMC11404265; doi:10.1136/bmjgast-2024-001483)
Supplement: online supplemental file 3 [file bmjgast-11-1-s003.pdf]

Theme	Quotes
Characteristics of IBD pain	*"UC and Crohn's disease are very different. Pain in UC is usually probably visceral hypersensitivity, IBS type symptoms, [...] so probably responds quite well to things like a low FODMAP diet. In Crohn's it's far more complex"* (CP1)
IBD patient as an individual	*"…doctors look at the result. Oh well, your tests are okay so you can't possibly be having all these problems and pain and symptoms, because your markers are not off. So you don't tend to get believed [...] they don't listen to the patient what the patient is telling them is no individualism at all. You're all grouped under the same umbrella" (PT2)*
Patient beliefs and experiences	*"… if I don't feel I'm getting what I need [...] I can be quite a bolshie patient. And I think that it shouldn't be that way, but that that is the experience that a lot of people have that you, you've got to fight for what you need"* (PT3)
Disease activity influencing symptoms and pain therapy choice	*"(...) I suppose the (...) slightly different issue is in those people who do have active inflammatory bowel disease but do have significant pain. We tend to get obsessed with treating the inflammation, and less good at treating the pain (CP1)."*
IBD treatment accessibility barriers	*"I do physiotherapy every 4-6 weeks because that's what's available in my area and that's what I could afford to pay for" (PT5)*<br><br>*"We do have dietary services, but they have a hell of a waiting list" (CP5).*
Patients seeking therapies privately	*"Personally, from my experience, I've been pretty much left to my own devices and, I seek out my own therapies,"* (PT5)<br><br>*"Private nutritionist [..] it costs but gives me good advice then measure nutrition levels vitamins, minerals etc [..] I do think it makes a huge difference, but it isn't something that's ever really been mentioned through the NHS"* (PT3)
Desirable MDT discussion	*"You know, it's a lot about talking to other people, collaborating to try to get the best care for the patients"* (CP3)
Pharmacological pain therapies	"*we're not allowed to take nonsteroidal anti-inflammatories, which, you know, do work very well for people who don't have inflammatory conditions. We are limited, basically, to paracetamol, which kind of doesn't work very well. Or coating with all sorts of side effects with it and then the other morphine based [drugs].*" (PT4)<br><br>"*In my experience, physicians are uncomfortable using tricyclics, (…) [as] of the 6 tricyclics that are available in the United States, one is not the same as the other, you have to try different ones, you have to make sure you are not getting side effects particularly the weight gain, (…) so it takes a lot of work and effort I think to use tricyclics as chronic pain.*" (CP5)
Cannabis use	*"(...) the pressure now on us now is that sometimes patients demand being prescribed cannabis and you just legally can't, and also there is no evidence that it helps, and also CBD oil, which mechanistically shouldn't do very much gets really hyped by patients." (CP4)*
Non-pharmacological pain therapies	"(...) *you try and deal with it yourself. Mind [you] if I can get to the gym and get in the pool, I am a happy bunny, because it helps me with my joint pains and my abdominal pain*" (PT1)<br><br>*"(…) it kind of gives you back some control as well and it's a non-medical intervention that I think we may be feeling a lot happier about it there's no kind of side effects or limited side effects involved with it (…)."* (PT3)
Low FODMAP diet	*"My patients who have persistent symptoms that sound as if they would otherwise be of irritable bowel type spectrum, and I am quite happy that their disease is well controlled, [low FODMAP diet] would be the first line therapy" (CP3)*<br><br>*"FODMAP diet if I am careful, actually does give me more control than anything else. But at the time, 20 odd years ago, I had to actually push to do it. But it did have a big effect" (PT5)*

Psychological therapies	*"[Mediation] gives me a calmer way to start the day and close the day I suppose it kind of resets so it's kind of waking up and hitting the day with your head fizzing it's like it's a mental reboot you just kind of have some calm time." (PT7)*<br><br>*"In theory, [hypnotherapy and cognitive behavioural therapy] are good targets but realistically unless you have access to a very specialized unit, which just happens to have a sort of a psychologist attached. I would think we're talking in the units in one hand probably they have that available." (CP2)*
Interactions between diet and psychological therapies	*"...my dietary habits and stress but also [other] patients [said] that the more stressed they are, the less they pay attention to their diet, all goes out of the window." (PT6)*<br><br>*"If you drive [disease activity] down you drive down the symptoms and you can equally drive down the anxiety because basically the things that people get anxious about are less likely to happen because they know they can get a control back." (CP2)*
Quality of life and functional outcomes	*"[I would aim for] improvement to make pain manageable so you can get on with your life, even though you've got chronic pain."* (PT8)<br><br>*"Can I get up and get dressed and go out and do things or do I need to stay in bed. Go to work. Or am I stuck at home because I need to be wired this way now. Can I go and see my friends or my family now that lockdown's over or am I in too much pain that is very much." (PT9)*
Research priorities	*"it's a bit idealistic to imagine that maybe pain would never, ever be there, even though we would all like that, but if you could make it a bit easier, it would help." (PT9)*<br><br>"*…for acute pain, I would agree with that, improvement in the intensity of pain, that should be number one. For chronic pain, my experience (..) is that intensity is not the problem, intensity is the same all the time. (…) So, with chronic pain, I would put improvement in the frequency of the pain*" (CP5)

Supplementary Table 13. Main themes and quotes identified during analysis

Supplementary comments

Characteristics of IBD pain

Pain intensity was another aspect of pain that was subjective but impactful on IBD patients' quality of life (QoL).

"...because I mean, I rarely ever go out to eat these days cause the number of times I've just been doubled over with really bad cramps to the point that I can't stand up straight and almost hobbled out of the restaurant. Quite honestly, it's embarrassing, you affect everybody else you are with. So, I just don't do those sorts of social things which are quite a big part of social life in non-covid times." (PT3)

"So, the first thing we would do is to figure out where the pain is coming from. Is it Sort of abdominal wall pain, is it related to movement of bowels. Is it not related to either of this? And your approach to dealing with it would be different for each of those. But if you have determined that you have got pain that's not related to that activity of your bowel and it's not in the abdominal wall, then you have got, you can call it hypersensitivity if you want" (CP2)

Many participants reported IBD medications can be effective in addressing pain intensity although those medications were unable to sustain their effects.

The capricious nature of IBD pain, coupled with its chronicity was highlighted:

"... I had a lot of pain in the lower abdomen [...] I had to just lie down in the foetal position, clench my teeth" (PT1).

Importantly, pain in IBD was not mentioned in isolation as pain tended to intricate or interplay with IBD-associated symptoms like fatigue and other comorbidities:

"I feel that your pain and fatigue go together because if you're in pain, you're fatigued, it's one, one bounces off the other" (PT2).

Participants (both patients and healthcare professionals) emphasised the need for better understanding of complexities surrounding IBD-pain which can vary considerably among IBD patients.

"...ulcerative colitis is that it's so wide ranging, that people can be on different ends of our ulcerative colitis spectrum, and they may experience completely different symptoms" (PT1).

"I think Crohn's and all IBD, there's such slippery conditions. And one of the things you hear repeatedly is different things work with different people and different people react in different ways and it's difficult to predict how something's going to work with one person or another" (PT1)

IBD patient as an individual

"...no 2 patients are the same" (CP2).

Patient beliefs and experiences

"They think you are some sort of neurotic hypochondriac [...] You are fobbed off, basically, so you stop going. You stop even asking anybody for help" (PT2).

There was also change in patients' behaviours when it came to accessing medical care. Patients started becoming firmer in their communication when requesting medical help and adapted the use of certain key words to successfully get an appointment. Understandably, patients fear this approach might make them appear as unpleasant patients. However, patients have only started using the approach due to increasing difficulty in accessing their usual medical care:

Variability in pain treatment approaches have been highlighted especially in using analgesia for pain control among IBD patients:

"I didn't want to become, you know, reliant on anything like that but it did seem to almost block it out after 3 days" (PT4).

Healthcare professionals had also expressed their reluctance when it comes to the use of medications in controlling IBD symptoms due to the medications potential side effects:

"I think we have realised how much damage we've done with the side effects and the interactions [...] actually a lot of them have contributed to a lot of the symptoms that there were left dealing with afterwards and it's a terrible cycle" (CP5)

Disease activity influencing symptoms and pain therapy choices

On the other hand, the latter group where IBD is inactive, but chronic pain is present, remains the most problematic to manage.

"(..) the more difficult ones are the ones where there is clearly persistent pain (..) where the disease appears to be otherwise, under good control and play (CP1)".

IBD treatment accessibility barriers

Health professionals expressed their dissatisfaction of the long waiting list of accessing psychological therapies for their patients by saying:

"Their view is that stress management, relaxation maybe possibly some cognitive behavioural therapy as vital focuses and some of the reasons they gave were quite wide ranging from lack of effectiveness of other options, the fact that other options weren't available, some people felt that maybe they're cost effective, some felt that there was an

element of postcode lottery coming through. Other people have been offered it, so why shouldn't they and one of the final things" (PT3)

"Yes, they're offering anything from cognitive behavioural therapy to distraction and breathing techniques advice. Because I work in London, we can refer to the Homeopathic hospital where they can do some hypnotherapy actually as well" (CP3)

"Acupuncture has helped me a lot, but I'm not saying it's cheap because it isn't" (PT4)

<u>Desirable MDT discussions</u>

On the other hand, if the MDT members do the opposite of the above and stop communicating with each other about the patient clinical status or ongoing IBD symptoms. This could result in unnecessarily delays to providing adequate patient care and might even cause harm of making late diagnosis of new presentation.

"You could have some completely different disease, but nobody will investigate that because they've put everything under that same label of Crohn's [...] I was in hospital recently with a really bad back and they said 'well, it's part of Crohn's', until they did the MRI, and they found I have a few discs impinging on the nerves" (PT5)

<u>Pharmacological pain therapies</u>

Healthcare professionals acknowledged that the pharmacological options for chronic pain are limited and ineffective.

"I think opiates are terrible drugs for chronic pain, they are good for acute pain, but they are really almost contraindicated in almost any chronic pain." (CP2)

<u>Non-pharmacological pain therapies</u>

On the one hand, patients favour these therapies, because the pharmacological treatments are limited in IBD, or can prove to be inefficient.

"*I think after a while we suppose patients get a bit fed up with just being given medicine after medicine and you know very often it doesn't work or it doesn't work well enough*" (PT2)

A unanimous belief in both patients and healthcare professionals is that there is lack of dietetic input in Adult IBD consultations and most patients either just self-exclude foods they cannot tolerate or seek advice privately.

"[*Diet] it's something that doctors typically don't sort of talk about and they tend to say to you that diet doesn't matter but I think instinctively as patients we sort of feel that it does and anecdotally, I've had a lot of people say that diets made a big differenc*e (…)" (PT4)

The low availability is thought to be due to low evidence, high costs and complexity, low compliance due to diet restrictions.

"*[Diets] are time consuming and expensive, and it's not just for the NHS, they're also expensive for patients as well as to try and figure out. Not just in terms of financial cost, but in terms of time as well and it can be restrictive." (CP3)*

<u>Quality of life and functional outcomes</u>

Medical professionals also believe that research should be aimed at ensuring patients have a good quality of life without pain.

"*What has to be a priority is into pain because, you know, the pain is the main issue, pain in the abdomen, pain in joints, (...) [and it] decreases your quality of life and this is the thing that patients need to try to establish a good quality of life with no pain*" (CP1)

<u>Research priorities</u>

However, some patients did raise the concern that even though having no pain is not achievable, they would ultimately still strive to have days when they can achieve all they planned for.

"*Why can't we have a day where we can go to the gym and we can go also go for a walk and we can go out for lunch. Well, just to do those basic things that everybody else does, why not? Why can't we?*" (PT10)

Other research goals mentioned by medical professionals were the effectiveness of low FODMAP diet, the distinction between the subtypes of IBD to be taken into consideration, brain gut axis and visceral hypersensitivity.

"[This is] where the research should be: how do I process my brain gut axis, how do I deal with visceral hypersensitivity, things like that." (CP6)
