# Supplementary material for: Patients’ and health professionals’ research priorities for chronic pain associated with inflammatory bowel disease: a co-produced sequential mixed methods Delphi consensus study
Source: BMJ Open Gastroenterol. 2024 Sep 12;11(1):e001483. doi: 10.1136/bmjgast-2024-001483 (PMC11404265; doi:10.1136/bmjgast-2024-001483)
Supplement: online supplemental file 4 [file bmjgast-11-1-s004.pdf]

University of Central Lancashire
UCLan

Pain in Crohn’s and Colitis. Online discussion events

Lead facilitator: Professor Morris Gordon

Ground Rules

Please no recording

Be respectful to your fellow participants

Participation is voluntary and you do not need to answer a question if you don't feel comfortable

There are no right or wrong answers. We want to hear your thoughts and opinions

Please try to avoid revealing any personally identifiable information (e.g. your full name, address, date of birth)

Please try to avoid naming specific doctors, nurses, or other healthcare professionals

Help protect others' privacy by not discussing details outside the group

Feel free to ask any questions. You can raise your hand or you can use the chatbox function

Please don't ask for any medical advice. If you have any concerns about your physical of mental health, please consult your doctor

University of Central Lancashire UCLan

Aims of the session

1) **Discuss which treatments you think should be prioritised for research into pain, and why?**

Prioritisation allows funders, researchers, and policy makers to focus on what is truly important to patients and professionals

2) To hear your views on hearing the thoughts of other patients / carers / healthcare professionals

Hearing perspectives from other people affected in Crohn's and Colitis may be helpful in allowing us to reach a joint decision

3) **To try and find a middle-ground or a consensus based on the viewpoints and opinions of the participants**

So we can produce a set of recommendations for funders and researchers to focus on for future research looking at pain in Crohn's and Colitis

University of Central Lancashire
UCLan

Results from the second survey

Thank you for taking the time and effort to complete the second survey.

This is what the collective voice of patients, carers, and healthcare professionals thought about priorities for research

Low FODMAP diet — 1st

Stress management course — 2nd

Relaxation therapy — 3rd

University of Central Lancashire
UCLan
1828

Results from the second survey

This is what the collective voice of patients, carers, and healthcare professionals thought about priorities for research outcome measures

Most important

Improvement in the intensity of pain

Having no pain at all

Improvement in the frequency of pain

Fewer days in which pain is present

Least important
