# Supplementary material for: Patients’ and health professionals’ research priorities for chronic pain associated with inflammatory bowel disease: a co-produced sequential mixed methods Delphi consensus study
Source: BMJ Open Gastroenterol. 2024 Sep 12;11(1):e001483. doi: 10.1136/bmjgast-2024-001483 (PMC11404265; doi:10.1136/bmjgast-2024-001483)
Supplement: online supplemental file 5 [file bmjgast-11-1-s005.pdf]

Patient & Carer

Start of Block: Consent Statement

Q4 Title of the research study: Exploring the views of patients and carers living with Crohn's, colitis, or inflammatory bowel disease.
Lead researcher: Professor Morris Gordon Thank you for taking the time to complete this survey. Before you start, please read through the following information. **By selecting ‘I agree, proceed’, you are agreeing to the below statements**. I confirm I have read and understood the information sheet dated 26/01/2020 for the above study. I have had the chance to consider the information and send questions to the research team. I am happy that my questions have been answered by the research team.
I understand that taking part in this research study involves an anonymous online survey. Once I have completed it, I will be asked whether I would be happy to take part in the next stages of this research study. This will include another anonymous online survey and an invite to a virtual event with the research team and other patients and carers. I understand that taking part is voluntary and that I am free to stop (withdraw) at any time without having to say why. I am free to decline to answer any question or series of questions. I am also free to decline taking part in the next stages of the study. If I withdraw from this study, any data collected before the withdrawal will be kept, but no further data will be collected from me. I understand that the information provided will be held securely and in line with data protection requirements at the University of Central Lancashire. By proceeding with the survey, I am agreeing to take part in this study.

End of Block: Consent Statement

Start of Block: About You

X→ X→

Q5 Which of the following best describes yourself (please select one answer)

- ◯ I have Crohn's Disease (1)
- ◯ I have Ulcerative Colitis (2)
- ◯ I have another form of Inflammatory Bowel Disease (3)
- ◯ I look after somebody with Crohn's Disease (5)
- ◯ I look after somebody with Ulcerative Colitis (6)
- ◯ I look after somebody with another form of Inflammatory Bowel Disease (7)

Display This Question:

If Which of the following best describes yourself (please select one answer) = I have another form of Inflammatory Bowel Disease

Q26 What other form of Inflammatory Bowel Disease (IBD) do you have?

____

Display This Question:

If Which of the following best describes yourself (please select one answer) = I look after somebody with another form of Inflammatory Bowel Disease

Q27 Which other form of Inflammatory Bowel Disease (IBD) does the person you provide care to have? *(please note, if you provide care for more than one person with Crohn's, Colitis or IBD, then please answer all questions in relation to one of these people)*

____

Display This Question:

If Which of the following best describes yourself (please select one answer) = I look after somebody with Crohn's Disease

Or Which of the following best describes yourself (please select one answer) = I look after somebody with Ulcerative Colitis

Or Which of the following best describes yourself (please select one answer) = I look after somebody with another form of Inflammatory Bowel Disease

Q7 What relation are you to the person you provide care to?

- ◯ Friend (1)
- ◯ Partner (2)
- ◯ Sibling (3)
- ◯ Parent (4)
- ◯ Son or daughter (5)
- ◯ Other family member (6)
- ◯ Other, within my role as a carer (7)

Display This Question:

If What relation are you to the person you provide care to? = Other family member

Q68 Please specify ...

____

Display This Question:

If What relation are you to the person you provide care to? = Other, within my role as a carer

Q69 Please specify ...

____

Display This Question:

If What relation are you to the person you provide care to? = Parent

Or What relation are you to the person you provide care to? = Other family member

Or What relation are you to the person you provide care to? = Other, within my role as a carer

Or What relation are you to the person you provide care to? = Sibling

Q70 Is the person you provide care to less than 18 years of age?

- ◯ Yes (1)
- ◯ No (2)

End of Block: About You

Start of Block: Length of disease

Display This Question:

If Which of the following best describes yourself (please select one answer) = I have Crohn's Disease

Or Which of the following best describes yourself (please select one answer) = I have Ulcerative Colitis

Or Which of the following best describes yourself (please select one answer) = I have another form of Inflammatory Bowel Disease

Q6 How long has it been since you received your diagnosis of Crohn's Disease, Colitis, or Inflammatory Bowel Disease?

- ◯ < 1 year (1)
- ◯ 1 - 3 years (2)
- ◯ 3 - 5 years (3)
- ◯ 5 - 10 years (4)
- ◯ > 10 years (5)

Display This Question:

If Which of the following best describes yourself (please select one answer) = I look after somebody with Crohn's Disease

Or Which of the following best describes yourself (please select one answer) = I look after somebody with Ulcerative Colitis

Or Which of the following best describes yourself (please select one answer) = I look after somebody with another form of Inflammatory Bowel Disease

Q14 How long has it been since the person you provide care to received their diagnosis of Crohn's, Colitis or Inflammatory Bowel Disease?

- ◯ < 1 year (1)
- ◯ 1 - 3 years (2)
- ◯ 3 - 5 years (3)
- ◯ 5 - 10 years (4)
- ◯ > 10 years (5)

End of Block: Length of disease

Start of Block: Frequency of Pain

Display This Question:

If Which of the following best describes yourself (please select one answer) = I have Crohn's Disease

Or Which of the following best describes yourself (please select one answer) = I have Ulcerative Colitis

Or Which of the following best describes yourself (please select one answer) = I have another form of Inflammatory Bowel Disease

Q81 Is your Crohn's, Colitis, or Inflammatory Bowel Disease in remission?

- ◯ Yes (1)
- ◯ No (2)

Display This Question:

If Which of the following best describes yourself (please select one answer) = I look after somebody with Crohn's Disease

Or Which of the following best describes yourself (please select one answer) = I look after somebody with Ulcerative Colitis

Or Which of the following best describes yourself (please select one answer) = I look after somebody with another form of Inflammatory Bowel Disease

Q82 For the person you provide care to, is their Crohn's, colitis, or inflammatory bowel disease in remission?

- Yes (1)
- No (2)

Display This Question:

If Which of the following best describes yourself (please select one answer) = I have Crohn's Disease

Or Which of the following best describes yourself (please select one answer) = I have Ulcerative Colitis

Or Which of the following best describes yourself (please select one answer) = I have another form of Inflammatory Bowel Disease

Q28 In the past 12 months, I have suffered with pain relating to my Crohn's, Colitis or Inflammatory Bowel Disease ....

- At least once a day (1)
- A few times a week (2)
- A few times a month (3)
- A few times a year (4)
- My IBD has not caused pain in the last 12 months (5)

Display This Question:

If Which of the following best describes yourself (please select one answer) = I look after somebody with Crohn's Disease

Or Which of the following best describes yourself (please select one answer) = I look after somebody with Ulcerative Colitis

Or Which of the following best describes yourself (please select one answer) = I look after somebody with another form of Inflammatory Bowel Disease

Q36 In the past 12 months, the person I provide care to has experienced pain relating to their Crohn's, Colitis of Inflammatory Bowel Disease ...

- ◯ At least once a day (1)
- ◯ A few times a week (2)
- ◯ A few times a month (3)
- ◯ A few times a year (4)
- ◯ Their IBD has not caused pain in the last 12 months (5)

Display This Question:

If Which of the following best describes yourself (please select one answer) = I have Crohn's Disease

Or Which of the following best describes yourself (please select one answer) = I have Ulcerative Colitis

Or Which of the following best describes yourself (please select one answer) = I have another form of Inflammatory Bowel Disease

Q71 In the past 12 months, pain relating to my Crohn's, Colitis or Inflammatory Bowel Disease, has impacted my quality of life ...

- ◯ 1 (insignificant) (1)
- ◯ 2 (minor) (2)
- ◯ 3 (moderate) (3)
- ◯ 4 (major) (4)
- ◯ 5 (in a very significant way) (5)

Display This Question:

If Which of the following best describes yourself (please select one answer) = I look after somebody with Crohn's Disease

Or Which of the following best describes yourself (please select one answer) = I look after somebody with Ulcerative Colitis

Or Which of the following best describes yourself (please select one answer) = I look after somebody with another form of Inflammatory Bowel Disease

Q72 In the past 12 months, the pain experienced by the person I provide care to, related to Crohn's, Colitis of inflammatory bowel disease, affects their quality of life ...

- ○ 1 (insignificant) (1)
- ○ 2 (minor) (2)
- ○ 3 (moderate) (3)
- ○ 4 (major) (4)
- ○ 5 (in a very significant way) (5)

End of Block: Frequency of Pain

Start of Block: Treatment for Pain (self)

Q29 We recently looked at completed research studies (such as clinical trials) which focus on treatments used for pain in people with Crohn's, Colitis or Inflammatory Bowel Disease. We've described the treatments below. Please share your experiences with each - you can tick more than one option.

Q36 **Low FODMAP Diet**
This is a diet of reduced fermentable carbohydrates in your food (FODMAPs). FODMAPs are types of fermentable carbodhydrate, which some people find hard to digest. A low FODMAP diet can help with symptoms such as abdominal pain, constipation, and diarrhoea in people with inactive Crohn's, Colitis or IBD.

- ☐ I have heard of this treatment to manage IBD pain (1)
- ☐ I have used this treatment to manage IBD pain (2)
- ☐ I have been recommended this treatment to manage IBD pain by a healthcare professional (3)
- ☐ ⊗ None of the above (4)

Q37 **Acupuncture**
Acupuncture is a treatment using thin metal needles to stimulate certain points in the body (called acupoints). This can cause the body to produce natural substances, such as pain-relieving endorphins.

- ☐ I have heard of this treatment to manage IBD pain (1)
- ☐ I have used this treatment to manage IBD pain (2)
- ☐ I have been recommended this treatment to manage IBD pain by a healthcare professional (3)
- ☐ ⊗ None of the above (4)

Q38 **Mindfulness**
Mindfulness is being aware of the present moment - paying attention to your thoughts and feelings and the world around you. This awareness can improve mental wellbeing.

- ☐ I have heard of this treatment to manage IBD pain (1)
- ☐ I have used this treatment to manage IBD pain (2)
- ☐ I have been recommended this treatment to manage IBD pain by a healthcare professional (3)
- ☐ ⊗ None of the above (4)

Q30 **Stress management course**
Courses focused on relaxation strategies (such as breathing exercises, biofeedback, visualisation techniques)

- ☐ I have heard of this treatment to manage IBD pain (1)
- ☐ I have used this treatment to manage IBD pain (2)
- ☐ I have been recommended this treatment to manage IBD pain by a healthcare professional (3)
- ☐ ⊗ None of the above (4)

Q39 **Enteric-released glyceryl trinitrate**
A medicine taken via the mouth which works specifically in the gut. It is thought to ease pain by widening the blood vessels in the abdomen.

☐ I have heard of this treatment to manage IBD pain (1)

☐ I have used this treatment to manage IBD pain (2)

☐ I have been recommended this treatment to manage IBD pain by a healthcare professional (3)

☐ ⊗None of the above (4)

Q40 **Ororinab**
This is a medication taken via the mouth, which acts on the cannabinoid receptor 2

☐ I have heard of this treatment to manage IBD pain (1)

☐ I have used this treatment to manage IBD pain (2)

☐ I have been recommended this treatment to manage IBD pain by a healthcare professional (3)

☐ ⊗None of the above (4)

Q41 **Relaxation training**
Training in relaxation techniques such as meditation, guided imagery, breathing exercises, and self-hypnosis.

- [ ] I have heard of this treatment to manage IBD pain (1)
- [ ] I have used this treatment to manage IBD pain (2)
- [ ] I have been recommended this treatment to manage IBD pain by a healthcare professional (3)
- [ ] ⊗None of the above (4)

Q42 **Online education to treat pain**
Information about the basis of pain in IBD. It can be delivered face-to-face, but usually, it is delivered online.

- [ ] I have heard of this treatment to manage IBD pain (1)
- [ ] I have used this treatment to manage IBD pain (2)
- [ ] I have been recommended this treatment to manage IBD pain by a healthcare professional (3)
- [ ] ⊗None of the above (4)

Q43 **Yoga to treat pain**
Yoga is an exercise that focuses on strength, flexibility, and breathing to boost wellbeing.

- ☐ I have heard of this treatment to manage IBD pain (1)
- ☐ I have used this treatment to manage IBD pain (2)
- ☐ I have been recommended this treatment to manage IBD pain by a healthcare professional (3)
- ☐ ⊗ None of the above (4)

Q44 **Transcranial direct current stimulation**
A wearable headset that uses electrical currents to stimulate specific parts of the brain

- ☐ I have heard of this treatment to manage IBD pain (1)
- ☐ I have used this treatment to manage IBD pain (2)
- ☐ I have been recommended this treatment to manage IBD pain by a healthcare professional (3)
- ☐ ⊗ None of the above (4)

Q45 **Kefir diet**
Kefir is a fermented probiotic drink - meaning it contains 'gut-friendly' bacteria. Drinking it aims to increase the good bacteria in the gut.

- [ ] I have heard of this treatment to manage IBD pain (1)
- [ ] I have used this treatment to manage IBD pain (2)
- [ ] I have been recommended this treatment to manage IBD pain by a healthcare professional (3)
- [x] None of the above (4)

Q46 **Stellate ganglion block**
An injection of local anaesthetic into the collection of nerves in the lower end of the neck. It aims to reduce pain signals travelling to and from the abdomen.

- [ ] I have heard of this treatment to manage IBD pain (1)
- [ ] I have used this treatment to manage IBD pain (2)
- [ ] I have been recommended this treatment to manage IBD pain by a healthcare professional (3)
- [x] None of the above (4)

Q73 **Deikenchuto**
A traditional Japanese herbal medicine made up of ginger, ginseng, and zanthoxylum fruit

- ☐ I have heard of this treatment to manage IBD pain (1)
- ☐ I have used this treatment to manage IBD pain (2)
- ☐ I have been recommended this treatment to manage IBD pain by a healthcare professional (3)
- ☐ ⊗ None of the above (4)

Q77
Cannabidiol *Cannabidiol (CBD) is an active ingredient in cannabis derived from the hemp plant. It is not a psychoactive - it does not get you "high"*

- ☐ I have heard of this treatment to manage IBD pain (1)
- ☐ I have used this treatment to manage IBD pain (2)
- ☐ I have been recommended this treatment to manage IBD pain by a healthcare professional (3)
- ☐ ⊗ None of the above (4)

Q47 Have you used any other treatments to manage pain associated with IBD?

- ○ Yes (1)
- ○ No (2)

Display This Question:

If Have you used any other treatments to manage pain associated with IBD? = Yes

Q34 If yes, please specify

____

End of Block: Treatment for Pain (self)

Start of Block: Treatment Use (Self)

Q66 You have said you've tried these treatments for IBD pain. Please tell us whether they helped your IBD pain.

Display This Choice:

If Low FODMAP Diet This is a diet of reduced fermentable carbohydrates in your food (FODMAPs). FODMA... = I have used this treatment to manage IBD pain

Display This Choice:

If Acupuncture Acupuncture is a treatment using thin metal needles to stimulate certain points in th... = I have used this treatment to manage IBD pain

Display This Choice:

If Mindfulness Mindfulness is being aware of the present moment - paying attention to your thoughts... = I have used this treatment to manage IBD pain

Display This Choice:

If We recently looked at completed research studies (such as clinical trials) which focus on treatme... =

Display This Choice:

If Enteric-released glyceryl trinitrate A medicine taken via the mouth which works specifically in t... = I have used this treatment to manage IBD pain

Display This Choice:

If Ororinab This is a medication taken via the mouth, which acts on the cannabinoid receptor 2 = I have used this treatment to manage IBD pain

Display This Choice:

If Relaxation training Training in relaxation techniques such as meditation, guided imagery, breathi... = I have used this treatment to manage IBD pain

Display This Choice:

If Online education to treat pain Information about the basis of pain in IBD. It can be delivered fa... = I have used this treatment to manage IBD pain

Display This Choice:

If Yoga to treat pain Yoga is an exercise that focuses on strength, flexibility, and breathing to bo... = I have used this treatment to manage IBD pain

Display This Choice:

If Transcranial direct current stimulation A wearable headset that uses electrical currents to stimu... = I have used this treatment to manage IBD pain

Display This Choice:

If Kefir diet Kefir is a fermented probiotic drink - meaning it contains 'gut-friendly' bacteria. Dr... = I have used this treatment to manage IBD pain

Display This Choice:

If Stellate ganglion block An injection of local anaesthetic into the collection of nerves in the lo... = I have used this treatment to manage IBD pain

Display This Choice:

If Deikenchuto A traditional Japanese herbal medicine made up of ginger, ginseng, and zanthoxylum fruit = I have used this treatment to manage IBD pain

Display This Choice:

If Cannabidiol Cannabidiol (CBD) is an active ingredient in cannabis derived from the hemp plant. I... = I have used this treatment to manage IBD pain

	No effect (1) (1)	2 (2)	3 (3)	4 (4)	Maximum effect (5) (5)
Display This Choice: If Low FODMAP Diet This is a diet of reduced fermentable carbohydrates in your food (FODMAPs). FODMA... = I have used this treatment to manage IBD pain Low FODMAP diet (1)	o	o	o	o	o
Display This Choice: If Acupuncture Acupuncture is a treatment using thin metal needles to stimulate certain points in th... = I have used this treatment to manage IBD pain Acupuncture (2)	o	o	o	o	o
Display This Choice: If Mindfulness Mindfulness is being aware of the present moment - paying attention to your thoughts... = I have used this treatment to manage IBD pain Mindfulness (3)	o	o	o	o	o
Display This Choice: If We recently looked at completed research studies (such as clinical trials) which focus on treatme... = Stress management (4)	o	o	o	o	o

Display This Choice: If Enteric-released glyceryl trinitrate A medicine taken via the mouth which works specifically in t... = I have used this treatment to manage IBD pain Enteric-released glyceryl trinitrate (5)	○	○	○	○	○
Display This Choice: If Ororinab This is a medication taken via the mouth, which acts on the cannabinoid receptor 2 = I have used this treatment to manage IBD pain Ororinab (6)	○	○	○	○	○
Display This Choice: If Relaxation training Training in relaxation techniques such as meditation, guided imagery, breathi... = I have used this treatment to manage IBD pain Relaxation training (7)	○	○	○	○	○
Display This Choice: If Online education to treat pain Information about the basis of pain in IBD. It can be delivered fa... = I have used this treatment to manage IBD pain Online education (8)	○	○	○	○	○
Display This Choice: If Yoga to treat pain Yoga is an exercise that focuses on strength, flexibility, and breathing to bo... = I have used this treatment to manage	○	○	○	○	○

IBD pain Yoga for pain (9)					
Display This Choice: If Transcranial direct current stimulation A wearable headset that uses electrical currents to stimu... = I have used this treatment to manage IBD pain Transcranial direct current stimulation (10)	○	○	○	○	○
Display This Choice: If Kefir diet Kefir is a fermented probiotic drink - meaning it contains 'gut-friendly' bacteria. Dr... = I have used this treatment to manage IBD pain Kefir diet (11)	○	○	○	○	○
Display This Choice: If Stellate ganglion block An injection of local anaesthetic into the collection of nerves in the lo... = I have used this treatment to manage IBD pain Stellate ganglion (12)	○	○	○	○	○
Display This Choice: If Deikenchuto A traditional Japanese herbal medicine made up of ginger, ginseng, and zanthoxylum fruit = I have used this treatment to manage IBD pain Daikenchuto (13)	○	○	○	○	○
Display This Choice: If Cannabidiol Cannabidiol	○	○	○	○	○

(CBD) is an active ingredient in cannabis derived from the hemp plant. I... = I have used this treatment to manage IBD pain
Cannabidiol (14)

End of Block: Treatment Use (Self)

Start of Block: Treatment for Pain (carer)

Q51 We recently looked at completed research studies (such as clinical trials) which focus on treatments used for pain in people with Crohn's, Colitis or Inflammatory Bowel Disease. We've described the treatments below. Please share your experiences with each - you can tick more than one option.

Q52 Low FODMAP Diet
This is a diet of reduced fermentable carbohydrates in your food (FODMAPs). FODMAPs are types of fermentable carbodhydrate, which some people find hard to digest. A low FODMAP diet can help with symptoms such as abdominal pain, constipation, and diarrhoea in people with inactive Crohn's, Colitis or IBD.

- ☐ I have heard of this treatment to manage IBD pain (1)
- ☐ The person I provide care to has used this treatment to manage IBD pain (2)
- ☐ The person I provide care to has been recommended this treatment to manage their pain by a healthcare professional (3)
- ☐ ⊗None of the above (4)

Q53 Acupuncture
Acupuncture is a treatment using thin metal needles to stimulate certain points in the body (called acupoints). This can cause the body to produce natural substances, such as pain-relieving endorphins.

- ☐ I have heard of this treatment to manage IBD pain (1)
- ☐ The person I provide care to has used this treatment to manage IBD pain (2)
- ☐ The person I provide care to has been recommended this treatment to manage their pain by a healthcare professional (3)
- ☐ ⊗ None of the above (4)

Q54 Mindfulness
Mindfulness is being aware of the present moment - paying attention to your thoughts and feelings and the world around you. This awareness can improve mental wellbeing.

- ☐ I have heard of this treatment to manage IBD pain (1)
- ☐ The person I provide care to has used this treatment to manage IBD pain (2)
- ☐ The person I provide care to has been recommended this treatment to manage their pain by a healthcare professional (3)
- ☐ ⊗ None of the above (4)

Q55 Stress management course
Courses focused on relaxation strategies (such as breathing exercises, biofeedback, visualisation techniques)

- ☐ I have heard of this treatment to manage IBD pain (1)
- ☐ The person I provide care to has used this treatment to manage IBD pain (2)
- ☐ The person I provide care to has been recommended this treatment to manage their pain by a healthcare professional (3)
- ☒ None of the above (4)

Q56 Enteric-released glyceryl trinitrate
A medicine taken via the mouth which works specifically in the gut. It is thought to ease pain by widening the blood vessels in the abdomen.

- ☐ I have heard of this treatment to manage IBD pain (1)
- ☐ The person I provide care to has used this treatment to manage IBD pain (2)
- ☐ The person I provide care to has been recommended this treatment to manage their pain by a healthcare professional (3)
- ☒ None of the above (4)

Q57 Ororinab
This is a medication taken via the mouth, which acts on the cannabinoid receptor 2

- ☐ I have heard of this treatment to manage IBD pain (1)
- ☐ The person I provide care to has used this treatment to manage IBD pain (2)
- ☐ The person I provide care to has been recommended this treatment to manage their pain by a healthcare professional (3)
- ☒ None of the above (4)

Q58 Relaxation training
Training in relaxation techniques such as meditation, guided imagery, breathing exercises, and self-hypnosis.

- ☐ I have heard of this treatment to manage IBD pain (1)
- ☐ The person I provide care to has used this treatment to manage IBD pain (2)
- ☐ The person I provide care to has been recommended this treatment to manage their pain by a healthcare professional (3)
- ☒ None of the above (4)

Q59 Online education to treat pain
Information about the basis of pain in IBD. It can be delivered face-to-face, but usually, it is delivered online.

- ☐ I have heard of this treatment to manage IBD pain (1)
- ☐ The person I provide care to has used this treatment to manage IBD pain (2)
- ☐ The person I provide care to has been recommended this treatment to manage their pain by a healthcare professional (3)
- ☒ None of the above (4)

Q60 Yoga to treat pain
Yoga is an exercise that focuses on strength, flexibility, and breathing to boost wellbeing.

- ☐ I have heard of this treatment to manage IBD pain (1)
- ☐ The person I provide care to has used this treatment to manage IBD pain (2)
- ☐ The person I provide care to has been recommended this treatment to manage their pain by a healthcare professional (3)
- ☒ None of the above (4)

Q61 Transcranial direct current stimulation
A wearable headset that uses electrical currents to stimulate specific parts of the brain

- [ ] I have heard of this treatment to manage IBD pain (1)
- [ ] The person I provide care to has used this treatment to manage IBD pain (2)
- [ ] The person I provide care to has been recommended this treatment to manage their pain by a healthcare professional (3)
- [ ] ⊗None of the above (4)

Q62 Kefir diet
Kefir is a fermented probiotic drink - meaning it contains 'gut-friendly' bacteria. Drinking it aims to increase the good bacteria in the gut.

- [ ] I have heard of this treatment to manage IBD pain (1)
- [ ] The person I provide care to has used this treatment to manage IBD pain (2)
- [ ] The person I provide care to has been recommended this treatment to manage their pain by a healthcare professional (3)
- [ ] ⊗None of the above (4)

Q63 Stellate ganglion block
An injection of local anaesthetic into the collection of nerves in the lower end of the neck. It aims to reduce pain signals travelling to and from the abdomen.

☐ I have heard of this treatment to manage IBD pain (1)

☐ The person I provide care to has used this treatment to manage IBD pain (2)

☐ The person I provide care to have been recommended this treatment to manage their pain by a healthcare professional (3)

☒ None of the above (4)

Q74 Deikenchuto
A traditional Japanese herbal medicine made up of ginger, ginseng, and zanthoxylum fruit

☐ I have heard of this treatment to manage IBD pain (1)

☐ The person I provide care to has used this treatment to manage IBD pain (2)

☐ The person I provide care to have been recommended this treatment to manage their pain by a healthcare professional (3)

☒ None of the above (4)

Q78

Cannabidiol *Cannabidiol (CBD) is an active ingredient in cannabis derived from the hemp plant. It is not a psychoactive - it does not get you "high"*

☐ I have heard of this treatment to manage IBD pain (1)

☐ The person I provide care to has used this treatment to manage IBD pain (2)

☐ The person I provide care to have been recommended this treatment to manage their pain by a healthcare professional (3)

☐ ⊗ None of the above (4)

Q64 Has the person you provide care to used any other treatments to manage pain associated with their IBD?

○ Yes (1)

○ No (2)

Display This Question:

If Has the person you provide care to used any other treatments to manage pain associated with their... = Yes

Q65 If yes, please specify

____

End of Block: Treatment for Pain (carer)

Start of Block: Treatment Use (Carer)

Q32 You have said the person you care for has tried these treatments for IBD pain. Please tell us whether they helped their IBD pain.

Display This Choice:

If Low FODMAP Diet This is a diet of reduced fermentable carbohydrates in your food (FODMAPs). FODMA... = The person I provide care to has used this treatment to manage IBD pain

Display This Choice:

If Acupuncture Acupuncture is a treatment using thin metal needles to stimulate certain points in th... = The person I provide care to has used this treatment to manage IBD pain

Display This Choice:

If Mindfulness Mindfulness is being aware of the present moment - paying attention to your thoughts... = The person I provide care to has used this treatment to manage IBD pain

Display This Choice:

If Stress management course Courses focused on relaxation strategies (such as breathing exercises, b... = The person I provide care to has used this treatment to manage IBD pain

Display This Choice:

If Enteric-released glyceryl trinitrate A medicine taken via the mouth which works specifically in t... = The person I provide care to has used this treatment to manage IBD pain

Display This Choice:

If Ororinab This is a medication taken via the mouth, which acts on the cannabinoid receptor 2 = The person I provide care to has used this treatment to manage IBD pain

Display This Choice:

If Relaxation training Training in relaxation techniques such as meditation, guided imagery, breathi... = The person I provide care to has used this treatment to manage IBD pain

Display This Choice:

If Online education to treat pain Information about the basis of pain in IBD. It can be delivered fa... = The person I provide care to has used this treatment to manage IBD pain

Display This Choice:

If Yoga to treat pain Yoga is an exercise that focuses on strength, flexibility, and breathing to bo... = The person I provide care to has used this treatment to manage IBD pain

Display This Choice:

If Transcranial direct current stimulation A wearable headset that uses electrical currents to stimu... = The person I provide care to has used this treatment to manage IBD pain

Display This Choice:

If Kefir diet Kefir is a fermented probiotic drink - meaning it contains 'gut-friendly' bacteria. Dr... = The person I provide care to has used this treatment to manage IBD pain

Display This Choice:

If Stellate ganglion block An injection of local anaesthetic into the collection of nerves in the lo... = The person I provide care to has used this treatment to manage IBD pain

Display This Choice:

If Deikenchuto A traditional Japanese herbal medicine made up of ginger, ginseng, and zanthoxylum fruit = The person I provide care to has used this treatment to manage IBD pain

Display This Choice:

If Cannabidiol Cannabidiol (CBD) is an active ingredient in cannabis derived from the hemp plant. I... = The person I provide care to has used this treatment to manage IBD pain

	No effect (1) (1)	2 (2)	3 (3)	4 (4)	Maximum effect (5) (5)
Display This Choice: If Low FODMAP Diet This is a diet of reduced fermentable carbohydrates in your food (FODMAPs). FODMA... = The person I provide care to has used this treatment to manage IBD pain Low FODMAP diet (1)	○	○	○	○	○
Display This Choice: If Acupuncture Acupuncture is a treatment using thin metal needles to stimulate certain points in th... = The person I provide care to has used this treatment to manage IBD pain Acupuncture (2)	○	○	○	○	○
Display This Choice: If Mindfulness Mindfulness is being aware of the present moment - paying attention to your thoughts... = The person I provide care to has used this treatment to manage IBD pain Mindfulness (3)	○	○	○	○	○
Display This Choice: If Stress management course Courses focused on relaxation strategies (such as breathing exercises, b... = The	○	○	○	○	○

person I provide care to has used this treatment to manage IBD pain Stress management (4)					
Display This Choice: If Enteric-released glyceryl trinitrate A medicine taken via the mouth which works specifically in t... = The person I provide care to has used this treatment to manage IBD pain Enteric-released glyceryl trinitrate (5)	○	○	○	○	○
Display This Choice: If Ororinab This is a medication taken via the mouth, which acts on the cannabinoid receptor 2 = The person I provide care to has used this treatment to manage IBD pain Ororinab (6)	○	○	○	○	○
Display This Choice: If Relaxation training Training in relaxation techniques such as meditation, guided imagery, breathi... = The person I provide care to has used this treatment to manage IBD pain Relaxation training (7)	○	○	○	○	○
Display This Choice: If Online education to treat pain Information about the basis of pain in IBD. It can be delivered fa... = The person I provide care to has used this treatment to manage IBD pain	○	○	○	○	○

Online education (8)					
Display This Choice: If Yoga to treat pain Yoga is an exercise that focuses on strength, flexibility, and breathing to bo... = The person I provide care to has used this treatment to manage IBD pain Yoga for pain (9)	○	○	○	○	○
Display This Choice: If Transcranial direct current stimulation A wearable headset that uses electrical currents to stimu... = The person I provide care to has used this treatment to manage IBD pain Transcranial direct current stimulation (10)	○	○	○	○	○
Display This Choice: If Kefir diet Kefir is a fermented probiotic drink - meaning it contains 'gut-friendly' bacteria. Dr... = The person I provide care to has used this treatment to manage IBD pain Kefir diet (11)	○	○	○	○	○
Display This Choice: If Stellate ganglion block An injection of local anaesthetic into the collection of nerves in the lo... = The person I provide care to has used this treatment to manage IBD pain Stellate ganglion (12)	○	○	○	○	○

Display This Choice: If Deikenchuto A traditional Japanese herbal medicine made up of ginger, ginseng, and zanthoxylum fruit = The person I provide care to has used this treatment to manage IBD pain Daikenchuto (13)	○	○	○	○	○
Display This Choice: If Cannabidiol Cannabidiol (CBD) is an active ingredient in cannabis derived from the hemp plant. I... = The person I provide care to has used this treatment to manage IBD pain Cannabidiol (14)	○	○	○	○	○

End of Block: Treatment Use (Carer)

Start of Block: Research Focus

Q22 If future research could focus on just **three** treatments for IBD pain, which **three** would you choose?

- [ ] Low FODMAP diet (1)
- [ ] Acupuncture (2)
- [ ] Mindfulness (3)
- [ ] Stress management (4)
- [ ] Ororinab (5)
- [ ] Relaxation training (6)
- [ ] Online education (7)
- [ ] Yoga for pain (8)
- [ ] Transcranial direct current stimulation (9)
- [ ] Kefir diet (10)
- [ ] Stellate ganglion (11)
- [ ] Enteric-released glyceryl trinitrate (12)
- [ ] Daikenchuto (13)
- [ ] Cannabidiol (14)

Q83 Are there any other treatments you think future research for IBD pain should focus on?

◯ Yes (1)

◯ No (2)

Display This Question:

If Are there any other treatments you think future research for IBD pain should focus on? = Yes

Q79 If yes, please specify ....

____

Display This Question:

If If If future research could focus on just three treatments for IBD pain, which three would you choose? q://QID22/SelectedChoicesCount Is Equal to 3

Carry Forward Selected Choices from "If future research could focus on just three treatments for IBD pain, which three would you choose?"

X→

Q50 Please tell us why you've chosen these three treatments for future IBD pain research.

- ◯ Low FODMAP diet (1) ____
- ◯ Acupuncture (2) ____
- ◯ Mindfulness (3) ____
- ◯ Stress management (4) ____
- ◯ Ororinab (5) ____
- ◯ Relaxation training (6) ____
- ◯ Online education (7) ____
- ◯ Yoga for pain (8) ____
- ◯ Transcranial direct current stimulation (9) ____
- ◯ Kefir diet (10) ____
- ◯ Stellate ganglion (11) ____
- ◯ Enteric-released glyceryl trinitrate (12) ____
- ◯ Daikenchuto (13) ____
- ◯ Cannabidiol (14) ____

Q80 You stated: '**${Q79/ChoiceTextEntryValue}**' as another treatment for future IBD pain research. Please tell us why you have stated this treatment.

____

End of Block: Research Focus

Start of Block: Outcomes

Q35
Researchers use lots of different definitions of 'success' when looking at whether a treatment works for IBD pain. We've listed some of these below. We want to know which means the most to you (or the person you care for).

Please rank these from **1 (most important) to 7 (least important)**
______ Improvement in the frequency of pain (1)
______ An improvement in the intensity of pain (2)
______ Fewer days in which pain is present (3)
______ Fewer days with moderate or severe pain (4)
______ A reduction in pain by at least 30% (5)
______ Having no pain at all (6)
______ Change in pain intensity from 'severe' pain to 'moderate' pain (7)

End of Block: Outcomes
