# Supplementary material for: Patients’ and health professionals’ research priorities for chronic pain associated with inflammatory bowel disease: a co-produced sequential mixed methods Delphi consensus study
Source: BMJ Open Gastroenterol. 2024 Sep 12;11(1):e001483. doi: 10.1136/bmjgast-2024-001483 (PMC11404265; doi:10.1136/bmjgast-2024-001483)
Supplement: online supplemental file 6 [file bmjgast-11-1-s006.pdf]

Professional

Start of Block: Default Question Block

Q1 Title of the research study: Exploring the views of patients and carers living with Crohn's, colitis, or inflammatory bowel disease.
Lead researcher: Professor Morris Gordon Thank you for taking the time to complete this survey. Before you start, please read through the following information. By selecting ‘I agree, proceed’, you are agreeing to the below statements. I confirm I have read and understood the information sheet dated 26/01/2020 for the above study. I have had the chance to consider the information and send questions to the research team. I am happy that my questions have been answered by the research team.
I understand that taking part in this research study involves an anonymous online survey. Once I have completed it, I will be asked whether I would be happy to take part in the next stages of this research study. This will include another anonymous online survey and an invite to a virtual event with the research team and other professionals, patients and carers. I understand that taking part is voluntary and that I am free to stop (withdraw) at any time without having to say why. I am free to decline to answer any question or series of questions. I am also free to decline taking part in the next stages of the study. If I withdraw from this study, any data collected before the withdrawal will be kept, but no further data will be collected from me. I understand that the information provided will be held securely and in line with data protection requirements at the University of Central Lancashire. By proceeding with the survey, I am agreeing to take part in this study.

End of Block: Default Question Block

Start of Block: About you

Q3 What is your healthcare role?

- ○ Doctor (consultant) (1)
- ○ Doctor (speciality trainee) (2)
- ○ Doctor (general practioner) (3)
- ○ Specialist nurse (4)
- ○ Other (5)

Display This Question:

If What is your healthcare role? = Doctor (consultant)

Or What is your healthcare role? = Doctor (speciality trainee)

Q4 Which speciality do you work in?

____

Display This Question:

If What is your healthcare role? = Specialist nurse

Q5 What are you a specialist nurse in?

____

Display This Question:

If What is your healthcare role? = Other

Q6 Please specify ...

____

End of Block: About you

Start of Block: Frequency and severity

Q22 How frequently do your patients ask for support in managing pain associated with their IBD

- ○ Never (1)
- ○ Infrequently (2)
- ○ Sometimes (3)
- ○ Often (4)
- ○ All the time (5)

End of Block: Frequency and severity

Start of Block: Treatment for Pain

Q7 We recently evaluated the published research looking at treatments used to treat pain in patients with IBD. For the treatments described below, please indicate your experiences with each.

Q8 Low FODMAP Diet
This is a diet of reduced fermentable carbohydrates in your food (FODMAPs). FODMAPs are types of fermentable carbodhydrate, which some people find hard to digest. A low FODMAP diet can help with symptoms such as abdominal pain, constipation, and diarrhoea in people with inactive Crohn's, Colitis or IBD.

- ▢ I have heard of this treatment to manage IBD pain (1)
- ▢ I have recommended this treatment to be used by my IBD patients (2)
- ▢ ⊗None of the above (3)

Q9 Acupuncture
Acupuncture is a treatment using thin metal needles to stimulate certain points in the body (called acupoints). This can cause the body to produce natural substances, such as pain-relieving endorphins.

- ▢ I have heard of this treatment to manage IBD pain (1)
- ▢ I have recommended this treatment to be used by my IBD patients (2)
- ▢ ⊗None of the above (3)

Q10 Mindfulness
Mindfulness is being aware of the present moment - paying attention to your thoughts and feelings and the world around you. This awareness can improve mental wellbeing.

- ☐ I have heard of this treatment to manage IBD pain (1)
- ☐ I have recommended this treatment to be used by my IBD patients (2)
- ☐ ⊗None of the above (3)

Q11 Stress management course
Courses focused on relaxation strategies (such as breathing exercises, biofeedback, visualisation techniques)

- ☐ I have heard of this treatment to manage IBD pain (1)
- ☐ I have recommended this treatment to be used by my IBD patients (2)
- ☐ ⊗None of the above (3)

Q12 Enteric-released glyceryl trinitrate
A medicine taken via the mouth which works specifically in the gut. It is thought to ease pain by widening the blood vessels in the abdomen.

- ☐ I have heard of this treatment to manage IBD pain (1)
- ☐ I have recommended this treatment to be used by my IBD patients (2)
- ☐ ⊗None of the above (3)

Q13 Ororinab
This is a medication taken via the mouth, which acts on the cannabinoid receptor 2

- ☐ I have heard of this treatment to manage IBD pain (1)
- ☐ I have recommended this treatment to be used by my IBD patients (2)
- ☐ ⊗ None of the above (3)

Q14 Relaxation training
Training in relaxation techniques such as meditation, guided imagery, breathing exercises, and self-hypnosis.

- ☐ I have heard of this treatment to manage IBD pain (1)
- ☐ I have recommended this treatment to be used by my IBD patients (2)
- ☐ ⊗ None of the above (3)

Q15 Online education to treat pain
Information about the basis of pain in IBD. It can be delivered face-to-face, but usually, it is delivered online.

- ☐ I have heard of this treatment to manage IBD pain (1)
- ☐ I have recommended this treatment to be used by my IBD patients (2)
- ☐ ⊗ None of the above (3)

Q16 Yoga to treat pain

Yoga is an exercise that focuses on strength, flexibility, and breathing to boost wellbeing.

- [ ] I have heard of this treatment to manage IBD pain (1)
- [ ] I have recommended this treatment to be used by my IBD patients (2)
- [x] None of the above (3)

Q17 Transcranial direct current stimulation

A wearable headset that uses electrical currents to stimulate specific parts of the brain

- [ ] I have heard of this treatment to manage IBD pain (1)
- [ ] I have recommended this treatment to be used by my IBD patients (2)
- [x] None of the above (3)

Q18 Kefir diet

Kefir is a fermented probiotic drink - meaning it contains 'gut-friendly' bacteria. Drinking it aims to increase the good bacteria in the gut.

- [ ] I have heard of this treatment to manage IBD pain (1)
- [ ] I have recommended this treatment to be used by my IBD patients (2)
- [x] None of the above (3)

Q19 Stellate ganglion block
An injection of local anaesthetic into the collection of nerves in the lower end of the neck. It aims to reduce pain signals travelling to and from the abdomen.

- [ ] I have heard of this treatment to manage IBD pain (1)
- [ ] I have recommended this treatment to be used by my IBD patients (2)
- [x] None of the above (3)

Q28 Deikenchuto
A traditional Japanese herbal medicine made up of ginger, ginseng, and zanthoxylum fruit

- [ ] I have heard of this treatment to manage IBD pain (1)
- [ ] I have recommended this treatment to be used by my IBD patients (2)
- [x] None of the above (3)

Q29
Cannabidiol *Cannabidiol (CBD) is an active ingredient in cannabis derived from the hemp plant. It is not a psychoactive - it does not get you "high"*

- [ ] I have heard of this treatment to manage IBD pain (1)
- [ ] I have recommended this treatment to be used by my IBD patients (2)
- [x] None of the above (3)

Q20 Have you used any other treatments to manage pain associated with IBD?

- ◯ Yes (1)
- ◯ No (2)

Display This Question:

If Have you used any other treatments to manage pain associated with IBD? = Yes

Q21 If yes, please specify

____

Q24 For the treatments you indicated you have recommended to your patients, how effective did you find them?

Display This Choice:

If Low FODMAP Diet This is a diet of reduced fermentable carbohydrates in your food (FODMAPs). FODMA... = I have recommended this treatment to be used by my IBD patients

Display This Choice:

If Acupuncture Acupuncture is a treatment using thin metal needles to stimulate certain points in th... = I have recommended this treatment to be used by my IBD patients

Display This Choice:

If Mindfulness Mindfulness is being aware of the present moment - paying attention to your thoughts... = I have recommended this treatment to be used by my IBD patients

Display This Choice:

If Stress management course Courses focused on relaxation strategies (such as breathing exercises, b... = I have recommended this treatment to be used by my IBD patients

Display This Choice:

If Enteric-released glyceryl trinitrate A medicine taken via the mouth which works specifically in t... = I have recommended this treatment to be used by my IBD patients

Display This Choice:

If Ororinab This is a medication taken via the mouth, which acts on the cannabinoid receptor 2 = I have recommended this treatment to be used by my IBD patients

Display This Choice:

If Relaxation training Training in relaxation techniques such as meditation, guided imagery, breathi... = I have recommended this treatment to be used by my IBD patients

Display This Choice:

If Online education to treat pain Information about the basis of pain in IBD. It can be delivered fa... = I have recommended this treatment to be used by my IBD patients

Display This Choice:

If Yoga to treat pain Yoga is an exercise that focuses on strength, flexibility, and breathing to bo... = I have recommended this treatment to be used by my IBD patients

Display This Choice:

If Transcranial direct current stimulation A wearable headset that uses electrical currents to stimu... = I have recommended this treatment to be used by my IBD patients

Display This Choice:

If Kefir diet Kefir is a fermented probiotic drink - meaning it contains 'gut-friendly' bacteria. Dr... = I have recommended this treatment to be used by my IBD patients

Display This Choice:

If Stellate ganglion block An injection of local anaesthetic into the collection of nerves in the lo... = I have recommended this treatment to be used by my IBD patients

Display This Choice:

If Deikenchuto A traditional Japanese herbal medicine made up of ginger, ginseng, and zanthoxylum fruit = I have recommended this treatment to be used by my IBD patients

Display This Choice:

If Cannabidiol Cannabidiol (CBD) is an active ingredient in cannabis derived from the hemp plant. I... = I have recommended this treatment to be used by my IBD patients

	No effect (1) (1)	2 (2)	3 (3)	4 (4)	Maximum effect (5) (5)	Unknown (6)
Display This Choice: If Low FODMAP Diet This is a diet of reduced fermentable carbohydrates in your food (FODMAPs). FODMA... = I have recommended this treatment to be used by my IBD patients Low FODMAP diet (1)	○	○	○	○	○	○
Display This Choice: If Acupuncture Acupuncture is a treatment using thin metal needles to stimulate certain points in th... = I have recommended this treatment to be used by my IBD patients Acupuncture (2)	○	○	○	○	○	○
Display This Choice: If Mindfulness Mindfulness is being aware of the present moment - paying attention to your thoughts... = I have recommended this treatment to be used by my IBD patients Mindfulness (3)	○	○	○	○	○	○
Display This Choice: If Stress management course Courses focused on relaxation strategies (such as breathing	○	○	○	○	○	○

exercises, b... = I have recommended this treatment to be used by my IBD patients Stress management (4)						
Display This Choice: If Enteric-released glyceryl trinitrate A medicine taken via the mouth which works specifically in t... = I have recommended this treatment to be used by my IBD patients Enteric-released glyceryl trinitrate (5)	○	○	○	○	○	○
Display This Choice: If Ororinab This is a medication taken via the mouth, which acts on the cannabinoid receptor 2 = I have recommended this treatment to be used by my IBD patients Ororinab (6)	○	○	○	○	○	○
Display This Choice: If Relaxation training Training in relaxation techniques such as meditation, guided imagery, breathi... = I have recommended this treatment to be used by my IBD patients Relaxation training (7)	○	○	○	○	○	○
Display This Choice: If Online education to treat pain Information about the basis of pain in IBD. It can be delivered fa... = I have recommended this treatment to be used by my IBD patients	○	○	○	○	○	○

Online education (8)						
Display This Choice: If Yoga to treat pain Yoga is an exercise that focuses on strength, flexibility, and breathing to bo... = I have recommended this treatment to be used by my IBD patients Yoga for pain (9)	○	○	○	○	○	○
Display This Choice: If Transcranial direct current stimulation A wearable headset that uses electrical currents to stimu... = I have recommended this treatment to be used by my IBD patients Transcranial direct current stimulation (10)	○	○	○	○	○	○
Display This Choice: If Kefir diet Kefir is a fermented probiotic drink - meaning it contains 'gut-friendly' bacteria. Dr... = I have recommended this treatment to be used by my IBD patients Kefir diet (11)	○	○	○	○	○	○
Display This Choice: If Stellate ganglion block An injection of local anaesthetic into the collection of nerves in the lo... = I have recommended this treatment to be used by my IBD patients Stellate ganglion (12)	○	○	○	○	○	○

Display This Choice: *If Deikenchuto A traditional Japanese herbal medicine made up of ginger, ginseng, and zanthoxylum fruit = I have recommended this treatment to be used by my IBD patients* Deikenchuto (13)	○	○	○	○	○	○
Display This Choice: *If Cannabidiol Cannabidiol (CBD) is an active ingredient in cannabis derived from the hemp plant. I... = I have recommended this treatment to be used by my IBD patients* Cannabidiol (14)	○	○	○	○	○	○

End of Block: Treatment for Pain

Start of Block: Research Focus

*

Q27 Please select the **three** treatments you think should be prioritised by the academic community for the treatment of pain associated with IBD?

- ☐ Low FODMAP diet (1)
- ☐ Acupuncture (2)
- ☐ Mindfulness (3)
- ☐ Stress management (4)
- ☐ Ororinab (5)
- ☐ Relaxation training (6)
- ☐ Online education (7)
- ☐ Yoga for pain (8)
- ☐ Transcranial direct current stimulation (9)
- ☐ Kefir diet (10)
- ☐ Stellate ganglion (11)

Q31 Are there any other treatments you think future research for IBD pain should focus on?

- ○ Yes (1)
- ○ No (4)

Display This Question:

If Are there any other treatments you think future research for IBD pain should focus on? = Yes

Q33 If yes, please specify ...

____

Carry Forward Selected Choices from "Please select the three treatments you think should be prioritised by the academic community for the treatment of pain associated with IBD?"

X→

Q29 For the **three treatments** you have selected as priorities, please explain your decision.

○ Low FODMAP diet (1) ____

○ Acupuncture (2) ____

○ Mindfulness (3) ____

○ Stress management (4) ____

○ Ororinab (5) ____

○ Relaxation training (6) ____

○ Online education (7) ____

○ Yoga for pain (8) ____

○ Transcranial direct current stimulation (9) ____

○ Kefir diet (10) ____

○ Stellate ganglion (11) ____

Q32 You stated: '**${Q33/ChoiceTextEntryValue}**' as another treatment for future IBD pain research. Please tell us why you have stated this treatment.

____

End of Block: Research Focus

Start of Block: Outcomes

Q31 We have found that researchers use lots of different definitions of 'success' when looking at whether a treatment works for a patient with IBD-associated pain. We are keen to understand which you believe is most meaningful to your patients with IBD.

Please rank these from **1 (most important) to 7 (least important).**

______ Improvement in the frequency of pain (1)
______ Improvement in the intensity of pain (2)
______ Fewer days in which pain is present (3)
______ Fewer days with moderate or severe pain (4)
______ A reduction in pain by at least 30% (5)
______ Having no pain at all (6)
______ Change in pain intensity from 'severe' pain to 'moderate' pain (7)

End of Block: Outcomes
